# Somatic Variation of T-Cell Receptor Genes Strongly Associate with HLA Class Restriction

**DOI:** 10.1371/journal.pone.0140815

**Published:** 2015-10-30

**Authors:** Paul L. Klarenbeek, Marieke E. Doorenspleet, Rebecca E. E. Esveldt, Barbera D. C. van Schaik, Neubury Lardy, Antoine H. C. van Kampen, Paul P. Tak, Robert M. Plenge, Frank Baas, Paul I. W. de Bakker, Niek de Vries

**Affiliations:** 1 Division of Genetics, Department of Medicine, Brigham and Women's Hospital, Harvard Medical School, Boston, Massachusetts, United States of America; 2 Program in Medical and Population Genetics, Broad Institute of Harvard and MIT, Cambridge, Massachusetts, United States of America; 3 Department of Clinical Immunology and Rheumatology, Laboratory for Experimental Immunology, Academic Medical Center, University of Amsterdam, Amsterdam, The Netherlands; 4 Laboratory for Genome Analysis, Academic Medical Center, University of Amsterdam, Amsterdam, The Netherlands; 5 Department of Clinical Epidemiology, Biostatistics and Bioinformatics, University of Amsterdam, Amsterdam, The Netherlands; 6 Department of Immunogenetics, Sanquin Diagnostic Services, Amsterdam, The Netherlands; 7 Department of Epidemiology, University Medical Center, Utrecht, The Netherlands; 8 Department of Medical Genetics, University Medical Center, Utrecht, The Netherlands; Universitá Cattolica del S. Cuore, ITALY

## Abstract

Every person carries a vast repertoire of CD4^+^ T-helper cells and CD8^+^ cytotoxic T cells for a healthy immune system. Somatic VDJ recombination at genomic loci that encode the T-cell receptor (TCR) is a key step during T-cell development, but how a single T cell commits to become either CD4^+^ or CD8^+^ is poorly understood. To evaluate the influence of TCR sequence variation on CD4^+^/CD8^+^ lineage commitment, we sequenced rearranged TCRs for both α and β chains in naïve T cells isolated from healthy donors and investigated gene segment usage and recombination patterns in CD4^+^ and CD8^+^ T-cell subsets. Our data demonstrate that most V and J gene segments are strongly biased in the naïve CD4^+^ and CD8^+^ subsets with some segments increasing the odds of being CD4^+^ (or CD8^+^) up to five-fold. These V and J gene associations are highly reproducible across individuals and independent of classical HLA genotype, explaining ~11% of the observed variance in the CD4^+^ vs. CD8^+^ propensity. In addition, we identified a strong independent association of the electrostatic charge of the complementarity determining region 3 (CDR3) in both α and β chains, where a positively charged CDR3 is associated with CD4^+^ lineage and a negatively charged CDR3 with CD8^+^ lineage. Our findings suggest that somatic variation in different parts of the TCR influences T-cell lineage commitment in a predominantly additive fashion. This notion can help delineate how certain structural features of the TCR-peptide-HLA complex influence thymic selection.

## Introduction

The differentiation of lymphoid progenitor cells into CD4^+^ helper and CD8^+^ cytotoxic T cells is a complex process essential for adaptive immunity, but many details remain elusive. Key steps include somatic rearrangement of V(ariable), D(iversity) and J(oining) gene segments encoding the T-cell receptor (TCR) and subsequent selection of T cells in the thymus to give rise to a mature T-cell repertoire. At a critical point during their development, double-positive CD4^+^CD8^+^ T-cell precursors (thymocytes) commit to express exclusively either the CD4 or CD8 co-receptor in a process called CD4^+^/CD8^+^ lineage commitment [1]. Although the molecular interaction between the TCR and human leukocyte antigen (HLA) molecules is considered to be critical for HLA class restriction [2–4], there has been no systematic analysis to investigate how TCR sequence variation affects T-cell fate.

The TCR is characterized by its combinatorial sequence diversity within the individual, making in-depth characterization of TCR variation challenging. Diversity in TCR repertoire emerges as a result of somatic rearrangements of 45 Vα and 49 Jα gene segments located on chr14q11.2 (which encode the α chain of the TCR), and 48 Vβ, 2 Dβ and 13 Jβ gene segments located on chr7q34 (which encode the β chain of the TCR). During VDJ recombination, V, D and J segments are selected at random and joined together in an imprecise manner to form rearranged genes for the α and β chains separately [5]. To characterize the TCR repertoire, we (and others) have developed efficient protocols based on next-generation sequencing [6–10].

Recently, a computational method was developed for estimating the relative proportions of CD4^+^ and CD8^+^ T cells on the basis of sequence features of the CDR3 region of the TCRβ gene [11]. In an extension of this approach, we characterized sequence patterns of both TCRα and TCRβ genes in CD4^+^ and CD8^+^ T cells collected from healthy donors. In our analysis of >230,000 unique TCR transcripts, we specifically sought to test whether variation in different parts of the TCR are associated with CD4^+^ vs. CD8^+^ status (also referred to as HLA class restriction) using a robust statistical approach.

## Results

We collected mononuclear cells from peripheral blood of 18 healthy unrelated individuals, and sorted T cells by flow cytometry to isolate naïve CD4^+^ and CD8^+^ T cells (Table A and B in S1 File). Using a previously described sequencing protocol [8, 12, 13], we sequenced the TCRβ chain repertoire of naïve T cells (327,019 transcripts), yielding 121,063 and 110,009 unique TCR β transcripts of CD4^+^ and CD8^+^ T cells, respectively (Table C in S1 File). In these data, we observed 41 of the 48 functional Vβ genes and all 13 functional Jβ genes.

**Fig 1:**
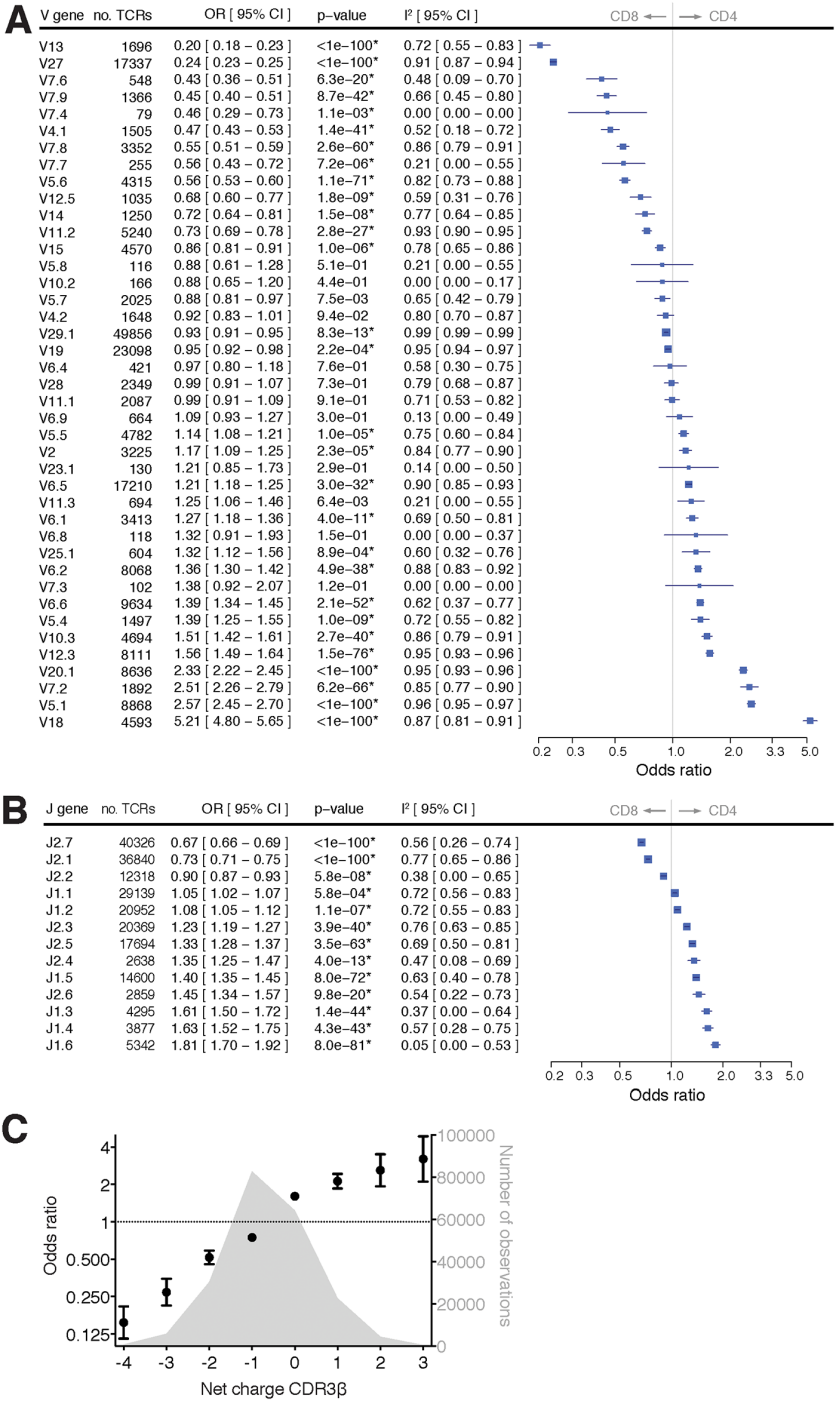
Impact of sequence variation of the TCR β chain on CD4^+^/CD8^+^ propensity. We analyzed 121,063 and 110,009 unique TCR β transcripts of naïve CD4^+^ and CD8^+^ T cells, respectively, from 18 healthy donors. The odds ratio (OR) is plotted for (**A**) each Vβ gene segment and (**B**) each Jβ gene segment, with OR < 1 indicating a propensity towards CD8^+^ and OR > 1 indicating a propensity towards CD4^+^. Total number of observations for each gene is listed. Significant associations after Bonferroni correction are denoted with an asterisk. (**C**) Odds ratios were computed for TCRs as a function of the calculated CDR3β net charge (error bars reflect 95% confidence intervals). A histogram of the number of observations is also plotted. Negative charge increases propensity of T cell towards CD8^+^ whereas positive charge increases propensity of T cell towards CD4^+^.

Because the Vβ chain is thought to make a direct interaction with the HLA [3, 4], we first tested each of the Vβ genes for association with CD4^+^/CD8^+^ status. Using a logistic regression model that adjusts for individual-specific effects and for the effects of the Jβ genes, we calculated odds ratios (ORs) for all individual Vβ gene segments (Fig 1A), where an OR > 1 indicates an increased propensity for CD4^+^ and an OR < 1 an increased propensity for CD8^+^. Across all observed Vβ genes, we observed a continuous gradient of the CD4^+^/CD8^+^ propensity, with ORs ranging from 0.2 (for Vβ13 being enriched in CD8^+^ T cells, p < 10^−100^) to 5.2 (for Vβ18 being enriched in CD4^+^ T cells, p < 10^−100^). Correcting for multiple testing (Bonferroni p < 0.0012), we found that 29 (60% of the 48 known Vβ genes) were statistically significantly associated with the likelihood of a T cell becoming either CD4^+^ or CD8^+^ (which we term here the CD4^+^/CD8^+^ propensity). Of the 12 Vβ genes that did not show a significant association, 6 had very few observations (<500 unique TCR transcripts), reducing power to detect an association. Although there was some heterogeneity between individuals (as indicated by the I^2^ metric; Fig 1A), the estimated magnitudes of effect between fixed and random effects models were very similar (see individual forest plots in S2 File). Furthermore, the associations remained highly statistically significant, even after adjusting for HLA genotype (Table D and Fig A in S1 File). Thus, the association of Vβ-genes with CD4^+^/CD8^+^ propensity is independent of the effect of each individual’s HLA genotype.

Next, we tested each of the Jβ gene segments for association with CD4^+^/CD8^+^ status, adjusting for individual donor effects and the effects of the Vβ genes. All 13 Jβ genes were significantly associated with effect sizes ranging from OR = 0.67 for Jβ2.7 (p < 10^−100^) to OR = 1.8 for Jβ1.6 (p < 10^−80^) (Fig 1B). Again, the results were highly robust across individuals (see individual forest plots in S3 File) and were independent of the effect of HLA genotypes (Fig B in S1 File).

These results show that Vβ and Jβ gene segments are distributed in a highly non-random fashion in the naïve CD4^+^ and CD8^+^ T-cell subsets. Adjusting for the influence of HLA genotype, individual Vβ genes (8.9%) and, to a lesser extent, Jβ genes (2.0%) are associated with CD4^+^ or CD8^+^ status, explaining effectively about 11% of the observed variance in CD4^+^/CD8^+^ numbers (Table 1). We further tested whether specific combinations of Vβ and Jβ gene segments could explain CD4^+^/CD8^+^ propensity beyond the effects of the Vβ and Jβ gene segments, but we found evidence for few such statistical interactions (Fig C and Table E in S1 File). The associations of Vβ and Jβ gene segment usage with CD4^+^/CD8^+^ propensity thus appear to be largely additive (Table 1).

**Table 1:** Percentage of CD4+/CD8+ propensity explained by V-genes, J-genes and CDR3-net charge^2^.

Variable(s)	Variance of CD4+/CD8+propensity explained (%)^1^
**Vβ genes**	8.9
**Jβ genes**	2.0
**Vβ + Jβ genes**	10.7
**CDR3β net charge**	3.3
**CDR3β length**	0.2
**Vβ + Jβ genes + CDR3β net charge**	13.1
**Vβ + Jβ genes + CDR3β net charge + CDR3β length**	13.1
**Vα genes**	9.6
**Jα genes**	2.0
**Vα + Jα genes**	11.2
**CDR3α net charge**	1.4
**CDR3α length**	0.4
**Vα + Jα genes + CDR3α net charge **	11.9
**Vα + Jα genes + CDR3α net charge + CDR3α length**	11.9

^1^Calculated as the difference between the Nagelkerke R^2^ of the null regression model (which only accounts for individual-specific effects) and the Nagelkerke R^2^ of an alternative model (which also includes the contribution of V and J genes, CDR3 net charge and/or CDR3 length).

^2^ Variation between different donors is shown in Table K in S1 File.

We also investigated those parts of the TCR thought to be most important for making direct contacts with cognate HLA and its bound peptide, namely the complementarity determining regions (CDRs) [3, 14]. We first focused on CDR3 as this is the most diverse part of the TCR due to the imprecise joining of the V, D and J segments (with CDR1 and CDR2 being entirely encoded by V genes). Thus, in contrast with Vβ and Jβ genes, sequence variation of the CDR3β is less straightforward to classify by canonical haplotypes that are encoded within the germline genome. Therefore, we took an alternative approach by counting the number of amino acids with charged, polar, aromatic and aliphatic side chains from the observed CDR3β transcripts. We also computed the net charge by subtracting the number of negatively charged amino acids (Asp, Glu) from the number of positively charged amino acids (Lys, Arg). After testing these CDR3β sequence features for association with CD4^+^/CD8^+^ status, we obtained the most significant results for net charge after correction for Vβ and Jβ genes (p < 10^−100^), which appears to have a quasi-linear effect on CD4^+^/CD8^+^ propensity (Fig 1C and Fig D in S1 File). The more positively charged the CDR3β segment, the more often it is observed in the CD4^+^ T-cell subset. Conversely, the more negatively charged the CDR3β segment, the more often it is observed in the CD8^+^ T-cell subset. Although CDR3β length was associated with CD4^+^/CD8^+^ propensity, the association disappeared after adjusting for Vβ/Jβ-genes and net charge (Table F and G in S1 File). These findings suggest a key role for an electrostatic interaction between TCR and HLA (and its bound peptide) in terms of committing a thymocyte towards the CD4^+^ or CD8^+^ lineage.

The emerging picture is that Vβ and Jβ have essentially independent associations with CD4^+^/CD8^+^ propensity with an additional association with CDR3β (Table 1). The CDR3β association accounts for about 3% of the variance in CD4^+^/CD8^+^ propensity (Table 1) but this is not entirely independent from the Jβ effect, because of limited overlap by a few amino acids (by convention) in the Jβ and CDR3β amino acid segments. More modest effects were observed for other motifs in CDR1β, CDR2β and CDR3β (Fig D and E in S1 File). Overall, these findings demonstrate significant differences between CD4^+^ and CD8^+^ T cells in terms of TCR sequence and amino acid composition.

**Fig 2:**
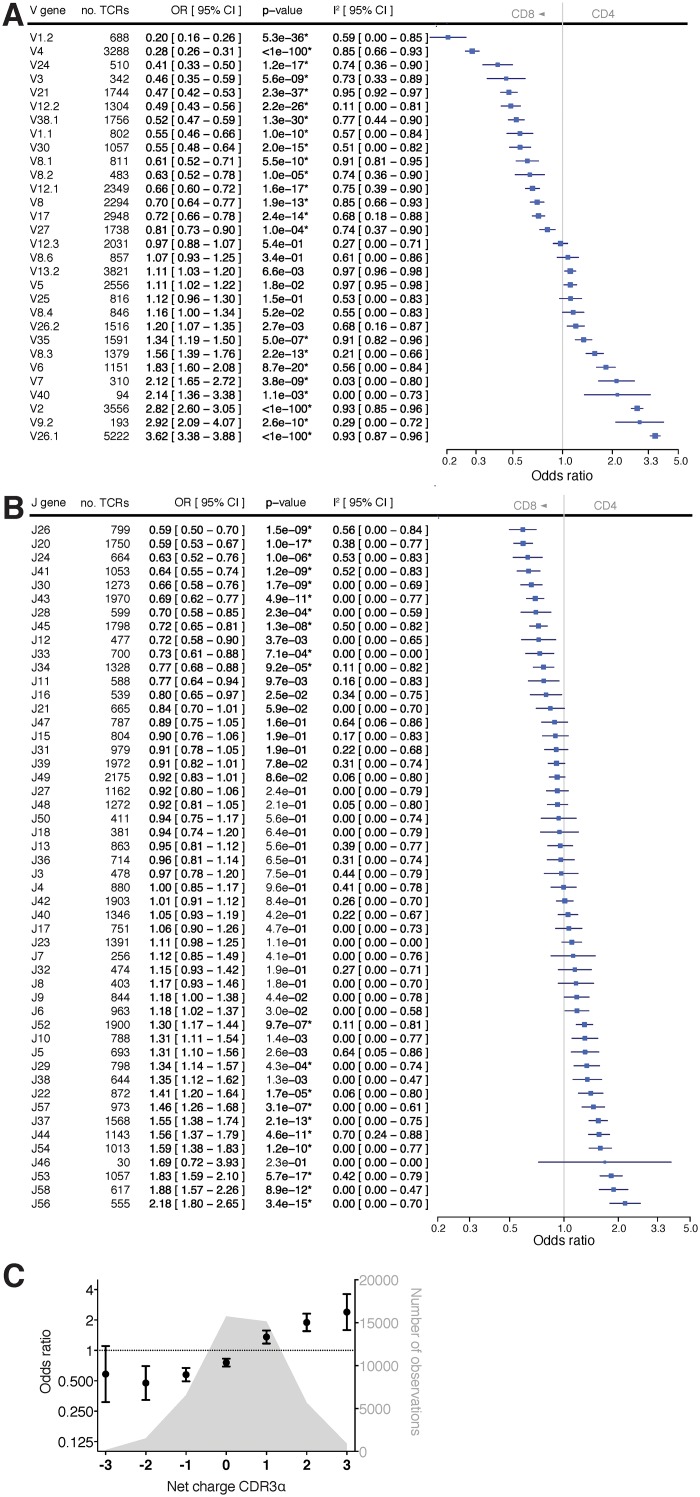
Impact of sequence variation of the TCR α chain on CD4^+^/CD8^+^ propensity. We analyzed 19,501 and 28,572 unique TCR α transcripts of naïve CD4^+^ and CD8^+^ T cells, respectively, from 5 healthy donors. The odds ratio (OR) is plotted for (**A**) each Vα gene segment and (**B**) each Jα gene segment, with OR < 1 indicating a propensity towards CD8^+^ and OR > 1 indicating a propensity towards CD4^+^. Total number of observations for each gene is listed. Significant associations after Bonferroni correction are denoted with an asterisk. (**C**) Odds ratios were computed for TCRs as a function of the calculated CDR3α net charge (error bars reflect 95% confidence intervals). A histogram of the number of observations is also plotted. Negative charge increases propensity of T cell towards CD8^+^ whereas positive charge increases propensity of T cell towards CD4^+^.

As the TCR β chain forms a heterodimer with the α chain we tested if similar effects would also be observed for the genes encoding the TCR α chain. To this end we sequenced 78,498 rearranged TCR α transcripts from 5 of the healthy donors (Table H in S1 File). Most Vα and Jα gene segments were associated with CD4^+^/CD8^+^ status (Fig 2), covering a similar range of effect sizes as observed for Vβ and Jβ genes. We found significant associations in 23 of 31 Vα genes (77%) and 20 significant associations in the 53 Jα genes (38%). Again, the associations were highly consistent across individuals. When we analyzed the amino acid motifs in CDR3α (Fig F and G in S1 File), we found a highly significant association for the net charge after correction for Vα and Jα genes (Table 1 and Fig 2C). Consistent with our observations for CDR3β above, a positive CDR3α charge was strongly associated with CD4^+^ T-cell fate while negative CDR3α charge was associated with CD8^+^. After adjusting for Vα and Jα genes and the CDR3α net charge, we also found an independent association for CDR3α length (Table I and J in S1 File and Table 1).

## Discussion

Overall, these findings reveal a surprising consistency in the extent to which variation in the α and β chains associate with CD4^+^/CD8^+^ propensity, with V genes explaining most of the effect (~9–10%). J genes appear to play a more modest role (~2%), while the net charge of the CDR3 explains another ~1–3% of the CD4^+^/CD8^+^ propensity (Table 1). Consistent with sights from crystal structures of the TCR-HLA complex [3, 4], both chains of the αβ TCR heterodimer appear to play important roles for HLA-peptide binding, each chain explaining a similar degree of the variance in CD4^+^/CD8^+^ propensity. The collective (additive) impact of V and J gene segments and CDR3 charge explains about 13% of the variance in CD4^+^/CD8^+^ propensity. Further, the contribution due to non-additive interactions of the V and J gene segments is modest considering all possible combinations. Because of technical limitations associated with single-cell sequencing at two separate chromosomal loci, we were unable to evaluate the cumulative effects of the α and β chains jointly; this will have to be addressed in the future.

Whether HLA class restriction is governed by motifs in the TCR germline has been debated fiercely in the past. Arguments against such motifs were typically based on the observation that all V and J genes were found in both CD4^+^ and CD8^+^ T-cell subsets [2, 4]. Secondly, crystallographic studies hinted at remarkable structural flexibility in TCR-HLA binding, even allowing a single TCR to recognize both an HLA class I and II complex [15]. On the other hand, there were early reports that several V and J genes exhibited a preference for either HLA class I or class II, although such preferences were often anecdotal and not generalizable for all V and J genes [4, 16–21]. Moreover, studying peripheral T cells in bulk potentially confounded many of these early studies. Through sequencing the TCR of large populations of isolated naïve T cells, we have taken a systematic and unbiased approach to address this question.

Recently, Emerson et al. reported that 21 out of 48 Vβ gene segments and 3 out of 13 Jβ gene segments exhibited differential usage in naïve CD4^+^ vs. CD8^+^ T cells [11]. In addition, they observed a substantial difference in CDR3 length between the two T- cell types. Here, we found that these associations are not limited to Vβ/Jβ genes but hold true for both α and β chains of the TCR. The percentages of genes reported here are probably underestimates as many of the genes were rare, limiting power to detect their association to CD4^+^/CD8^+^ propensity. It is worth highlighting that the associations found are highly consistent across unrelated individuals.

Although transcriptional and epigenetic changes in committed CD4^+^ and CD8^+^ T cells have been described in detail [22, 23], it is not clear if such changes reflect causal factors that truly drive lineage commitment or reflect changes as a result of the CD4^+^ vs. CD8^+^ commitment. Assuming that the somatic rearrangements of VDJ recombination are random and irreversible events that occur before lineage commitment, it is likely that the associations described here reflect a causal effect on thymic selection (as opposed to resulting from confounding phenomena). We did not, however, address the potential influence of homeostatic proliferation of naïve T cells. In theory the availability of self-peptides during homeostatic proliferation could bias the TCR repertoire, but it is unlikely that this could explain our findings. First, the associations were highly consistent across unrelated individuals across different age groups, while homeostatic proliferation plays a much more prominent role in elderly patients with a declining production of naïve T cells [24]. Secondly, all individuals in our cohort had different HLA genotypes. Thus, to the extent that homeostatic proliferation plays a role, its effect would be limited to introducing (random) noise, thereby reducing precision, but not result in a systematic skewing of CD4^+^/CD8^+^ numbers for specific TCR variants.

Our analysis demonstrates that somatic rearrangements of the αβ TCR are strongly associated with the propensity of a thymocyte to commit to the CD4^+^ or CD8^+^ lineage. It is important to emphasize that these relations are probabilistic—not deterministic. Moreover, the observed associations are largely additive; multiple motifs can thus influence the propensity of the individual T cell to a certain fate. It is worth noting that the absolute magnitude of effect for the individual TCR variants and the cumulative explained variance is quite high compared to other complex (multifactorial) phenotypes studied through genome-wide association analysis [25, 26], but not inconsistent with what is typically observed for HLA associations (for example, [27]). Other factors including HLA genotype and nature of the peptide presented are also likely to play a role. We hypothesize that expanding the dataset to a much larger sample (e.g. 1000) should provide sufficient statistical power to evaluate the quantitative influence of HLA genotype on CD4^+^/CD8^+^ propensity.

Our findings have important implications for studying the structural determinants that govern the ternary TCR-peptide-HLA complex. In particular, our results highlight an important role for the electrostatic properties of the CDR3 loop. Whereas the CDR1 and CDR2 loops are generally considered to make contacts with the HLA molecule, the CDR3’s primary interaction is thought to be with the peptide [3]. Assuming the charged groups on the CDR3 loop interact with the peptide, there may be a reciprocal effect due to the charge of the peptide, but this will need to be tested.

## Methods

### Ethics statement & buffy coats

Buffy coats were procured from blood donated by anonymous healthy blood donors at Sanquin Blood Supply, Amsterdam (blood donation center), the Netherlands (www.sanquin.nl). All donors provided written informed consent for use of leftover blood products for scientific research in accordance with the protocol of the local institutional review board, the Medical Ethics Committee of Sanquin Bloodbank (Amsterdam, The Netherlands) in accordance with the Declaration of Helsinki. As the buffy coats are leftover products from blood donation, no addition approval is required by Dutch law or by the local Medical Ethics Committee, the Medical Ethics Committee of Academic Medical Center-University of Amsterdam (Amsterdam, The Netherlands). Information on age, gender and HLA background is described in Table A and Table B in S1 File.

### HLA typing

Genomic DNA was isolated from peripheral blood mononuclear cells using the Gentra Autopure LS system and Gentra Puregene Blood Kit according to manufacturers protocols (Qiagen, Hilden, Germany). The DNA samples were typed at HLA-A, -B, and -C (exons 2, 3, 4) and -DRB1, -DQB1, -DPB1 (exons 2, 3). Sequencing reactions were performed according to the SBTexcellerator protocol (GenDX, Utrecht, the Netherlands) and subsequently loaded on an ABI3130xl Genetic Analyzer. The data was analyzed with the SBTengine software (GenDX, Utrecht, the Netherlands).

### Cell sorting

From the buffy coats, Peripheral Blood Mononuclear Cells (PBMCs) were isolated and prepared for cell sorting as previously described [12]. Cells for FACS sorting were stained in V-bottom microplates (cat no. 561101, Greiner Bio-one, Frickenhausen Germany) using the following antibodies: CD4-PerCP-cy5.5 (clones RPA-T4), CD8-PE-cy7 (clone SK1), CD27-APC (clone O323), CD45RA-FITC (HI100) and CD45RO-PE (UCHL1) (all eBiosciences, Vienna, Austria). Naïve CD4^+^ T cells were characterized as CD4+CD8−CD45RO−CD45RA+; Naïve CD8+ T cells as CD8^+^CD4-CD45RA^+^CD27bright. All sorted populations contained >5 x 10^5^ cells and were over >95% pure as confirmed by FACS analysis.

### Next-generation sequencing and bioinformatics

Linear amplification and next-generation sequencing (NGS) were performed as previously described [8, 12], starting from 500 ng of total RNA. NGS was performed on the Roche/454 Genome Sequencer using the Titanium platform. For the TCR α sequencing a set of primers was developed covering all functional TCR α gene variants as described by IMGT [28]. Primer sequences are available upon request. The protocol for preparation and NGS TCR α was identical to that of the TCR β analysis.

### Statistical analysis

Datasets were analyzed using R version 2.13.0 (http://www.R-project.org). All unique lymphocyte antigen receptor sequences were determined based on their unique VDJ junction (CDR3) according to IMGT nomenclature [29]. Duplicate transcripts were removed from the analysis. Bonferroni correction was applied for multiple testing. We used a logistic regression model with CD4^+^ vs. CD8^+^ status of each T cell as the dependent variable (outcome) and V and J genes as independent variables, while correcting for individual donor effects (R function ‘glm’, package ‘stats’). The heterogeneity index I^2^ was calculated as described in [30] (functions ‘metagen’ and ‘summary.meta’, package ‘meta’). Associations for individual V and J genes were also calculated per donor using the model described above. Fixed and random effect summaries were calculated using the function ‘metagen’ (package ‘meta’). We also computed standardized Z scores (beta coefficient divided by standard error) using the model described above (function ‘glm’, package ‘stats’). The influence of HLA variation was analyzed by including each observed HLA genotype into the model as a binary variable (carrier or non-carrier), restricting our analysis to the 14 HLA alleles present in at least 5 donors. We also tested for interactions between Vβ and Jβ genes, while correcting for individual donor effects. Based on the above models, we also determined Akaike Information Criterion (AIC) values using the ‘glm’ function. In all analyses, the AIC values of a model were compared to the AIC of the “null” model, which included only indicator variables to adjust for individual donor effects. We also report the variance explained as the Nagelkerke’s R^2^ from the logistic regression model (minus that of the null model).

## Supporting Information

S1 File Supplementary figures and results. (DOCX)

S2 File Individual forest plots Vβ-genes. (PDF)

S3 File Individual forest plots Vα-genes. (PDF)
