# Supplementary material for: Somatic Variation of T-Cell Receptor Genes Strongly Associate with HLA Class Restriction
Source: PLoS One. 2015 Oct 30;10(10):e0140815. doi: 10.1371/journal.pone.0140815 (PMC4627806; doi:10.1371/journal.pone.0140815)
Supplement: S1 File — (DOCX) [file pone.0140815.s001.docx]

**Supplementary online material**

**Somatic variation of T-cell receptor genes strongly associate with HLA class restriction**

Paul L. Klarenbeek^1-4^, Marieke E. Doorenspleet^3-4^, Rebecca E.E. Esveldt^3^,
Barbera D.C. van Schaik^5^, Neubury Lardy^6^, Antoine H.C. van Kampen^5^, Paul P. Tak^3,#a,#b^, Robert M. Plenge^1,2,#c^, Frank Baas^4^, Paul I.W. de Bakker^1,2,7,8*¶^, Niek de Vries^3¶^

^1^ Division of Genetics, Department of Medicine, Brigham and Women's Hospital, Harvard Medical School, Boston, Massachusetts, USA.

^2^ Program in Medical and Population Genetics, Broad Institute of Harvard and MIT, Cambridge, Massachusetts, USA.

^3^ Department of Clinical Immunology and Rheumatology, Laboratory for Experimental Immunology, Academic Medical Center, University of Amsterdam, Amsterdam, The Netherlands.

^4^ Laboratory for Genome Analysis, Academic Medical Center, University of Amsterdam, Amsterdam, The Netherlands.

^5^ Department of Clinical Epidemiology, Biostatistics and Bioinformatics, University of Amsterdam, Amsterdam, The Netherlands.

^6^ Department of Immunogenetics, Sanquin Diagnostic Services, Amsterdam, The Netherlands.

^7^ Department of Epidemiology, University Medical Center, Utrecht, The Netherlands.

^8^ Department of Medical Genetics, University Medical Center, Utrecht, The Netherlands.

^#a^ Currently: GlaxoSmithKline, Stevenage, United Kingdom

^#b^ University of Cambridge, Cambridge, United Kingdom

^#c^ Currently: Merck and Co., Boston, USA.

^¶^ Both authors contributed equally

**Content:**

Supplementary results (Figures and tables),

Please note that individual plots for per donor analyses for V-genes are attached as separate files

**Additional results**

**Additional information on the donor panel: Table A** shows the information on age and gender. Donors were collected to represent different age groups. **Table B** shows the 4 digit HLA-typing of all the donors. For 1 donor, no DNA was available for DNA analysis.

**Table A: Donor characteristics**

| Donors included | n=18 |
| --- | --- |
| Age-group (20-25yr) | n=6 |
| Age-group (40-50yr) | n=6 |
| Age-group (60-70yr) | n=6 |
| Mean age (range) | 44 (20-69) |
| Percentage female | 50% |

**Table B: Individual HLA genotypes**

| Donor | HLA-A | | HLA-B | | HLA-C | | HLA-DR | | HLA-DQ | | HLA-DP | |
| --- | --- | --- | --- | --- | --- | --- | --- | --- | --- | --- | --- | --- |
| 1 | NA | NA | NA | NA | NA | NA | NA | NA | NA | NA | NA | NA |
| 2 | 01:01 | 02:01 | 55:01 | 57:01 | 03:01 | 06:02 | 07:01 | 11:01 | 03:03 | 03:01 | 04:01/105:01 | 04:01/126:01 |
| 3 | 01:01 | 02:01 | 08:01 | 41:01 | 07:01/07:06/07:18 | 17:01/17:03 | 03:01 | 07:01 | 02:01 | 03:03 | 01:01 | 14:01 |
| 4 | 24:02 | 32:01 | 08:01 | 18:01 | 07:01/07:06/07:18 | 05:01 | 03:01 | 09:01 | 03:01 | 03:03 | 02:01 | 02:01 |
| 5 | 03:01 | 68:01 | 07:02/07:61 | 13:02 | 07:02/07:50 | 06:02 | 3:01 | 7:01 | 2:01 | 2:02 | 1:01 | 4:02 |
| 6 | 01:01 | 01:01 | 08:01 | 13:02 | 07:01/07:06/07:18 | 06:02 | 3:01 | 07:01 | 02:01 | 02:02 | 01:01 | 04:02 |
| 7 | 02:01 | 24:02 | 08:01 | 57:01 | 07:01/07:06/07:18 | 03:01 | 07:01 | 02:01 | 03:03 | 01:01 | 10:01 | 03:01 |
| 8 | 03:01 | 30:04 | 07:02/07:61 | 51:08 | 07:02/07:50 | 16:02 | 13:02 | 15:01 | 06:09 | 06:02 | 04:01 | 04:01 |
| 9 | 01:01 | 02:01 | 08:01 | 40:01 | 07:01/07:06/07:18 | 03:04 | 04:04 | 13:02 | 03:02 | 06:04 | 03:01 | 04:01 |
| 10 | 23:01/  23:17 | 32:01 | 40:02 | 44:03 | 15:02 | 04:01/04:82 | 07:01 | 14:54 | 02:02 | 05:03 | 04:01 | 04:01 |
| 11 | 01:01 | 24:02 | 08:01 | 15:17 | 07:01/07:06/07:18 | 11:01 | 15:01 | 03:01 | 06:02 | 01:01 | 04:01 | 11:01 |
| 12 | 24:02 | 24:03 | 07:02/  07:61 | 35:01/  35:42 | 07:02/07:50 | 04:01/04:82 | 11:01 | 15:01 | 03:01 | 06:02 | 02:01 | 04:01 |
| 13 | 02:01 | 02:01 | 07:02/07:61 | 41:01 | 07:02 | 07:01 | 03:01 | 15:01 | 02:01 | 06:02 | 04:01 | 04:01 |
| 14 | 03:01 | 11:01 | 07:02/07:61 | 37:01 | 07:02/07:50 | 06:02 | 04:01 | 10:01 | 03:01 | 05:01 | 02:01 | 19:01 |
| 15 | 02:01 | 24:162 | 15:01 | 44:02 | 04:01/04:82 | 05:01 | 03:01 | 04:01 | 02:01 | 03:02 | 04:02 | 09:01 |
| 16 | 01:01 | 11:01 | 51:01 | 55:01 | 15:02 | 03:03 | 03:01 | 14:54 | 02:01 | 05:03 | 01:01 | 04:01 |
| 17 | 03:01 | 23:01/  23:17 | 07:02/07:61 | 44:03 | 07:02/07:50 | 04:01/04:82 | 07:01 | 15:01 | 02:02 | 06:02 | 01:01 | 04:01 |
| 18 | 01:01 | 03:01 | 07:02/07:61 | 08:01 | 07:02 | 07:01 | 03:01 | 15:01 | 02:01 | 06:02 | 04:01 | 09:01 |

**Additional NGS-results on TCRß: Table C** shows the amount of reads and unique TCRß rearrangements (clones) that were available from the TCRß sequencing.

**Table C: TCRß-analysis: Number of NGS-reads and clones available for analysis**

|  | CD4 naïve | CD8 naïve |
| --- | --- | --- |
| Reads/donor* | 8,590 (6,357 - 11,108) | 8,190 (6,277 - 9,768) |
| Clones/donor** | 6,726 (3,723 - 10,461) | 6,112 (1,608 - 11,890) |
| Total clones | 121,063 | 110,009 |

* reads recovered after bioinfomatics; mean(range)

** clones detected; mean(range)

**Additional results on the influence of HLA on the association of Vß and Jß genes with CD4^+^/CD8^+^ propensity:** As HLA-alleles encode differences in HLA-structure, they are an intuitive factor to influence CD4^+^/CD8^+^ propensity. Although this study was not designed to understand the influence of HLA on CD4^+^/CD8^+^ propensity, we wanted to investigate whether HLA-alleles influenced our findings. Beforehand, the chance of an influence of HLA-alleles was very small as the model used in **Fig. 1** to determine the associations of Vß and Jß genes with CD4^+^/CD8^+^ propensity included a correction for interdonor variation. This correction automatically corrects for any HLA-influence.

To investigate the potential influence of HLA-alleles we performed the following analysis. We investigated whether correction for the presence of an HLA-allele (comparing carriers to non-carriers) would alter the significance of our findings in **Fig. 1** (correction for all interdonor variation). Please note that the latter is a much more stringent correction. To this end we selected the 14 HLA-alleles that were present in at least 5 donors (**Table D**). We found similar Z-scores when correcting for interdonor variation or for the presence of individual HLA-alleles (**Fig. A and B**). Therefore, HLA-variation in our donor-panel does not seem to bias our results, although an influence of HLA in general on CD4^+^/CD8^+^ propensity cannot be excluded based on these results.

**Table D: HLA-alleles present in 5 or more donors**

| HLA-genes | Alleles |
| --- | --- |
| HLA-A | 01:01, 02:01 |
| HLA-B | 07:02/07:61*, 08:01 |
| HLA-C | 07:01/07:06/07:18*, 07:02/07:50* |
| HLA-DR | 03:01, 07:01, 15:01 |
| HLA-DQ | 02:01, 03:01, 06:02 |
| HLA-DP | 01:01, 04:01 |

* Please note that these alleles are based on 4-digit typing. In these cases with * a further classification cannot be made based on 4-digit typing.

**Figure A: Addressing potential influence of HLA-alleles on associations between Vß genes and CD4^+^/CD8^+^-propensity.** For each Vß gene the original association is shown (blue). In red, 14 additional analyses where performed where we controlled for an individual HLA-allele (**Table D**). Please note that the 14 individual red dots overlap, so that it seems that there are only one or two. As shown the Z-score of the Vß gene associations with CD4^+^/CD8^+^-propensity when controlling for individual HLA-alleles is similar to that when controlling for all interdonor variation. Therefore, the presence of HLA-alleles does not seem to bias the associations between Vß genes and CD4^+^/CD8^+^-propensity described in the main text.

**Figure B: Addressing potential influence of HLA-alleles on associations between Jß genes and CD4^+^/CD8^+^-propensity.** For each Jß gene the original association is shown (blue). In red, 14 additional analyses where performed where we controlled for an individual HLA-allele (**Table D**). Please note that the 14 individual red dots overlap, so that it seems that there is only one or two. As shown the Z-score of the Jß gene associations with CD4^+^/CD8^+^-propensity when controlling for individual HLA-alleles is similar to that when controlling for all interdonor variation. Therefore, the presence of HLA-alleles does not seem to bias the associations found between Jß genes and CD4^+^/CD8^+^-propensity.

**Additional results on potential interaction of Vß and Jß genes:** As the TCRß-chain is a 3D-structure, interactions of Vß and Jß genes are a potential bias for the results described in **Fig. 1** of the main text. Although the model used in **Fig. 1** of the main text did already correct for Jß gene variation in the Vß genes analysis and vice versa, it did not correct for interactions between individual Vß and Jß genes. To investigate this potential source of bias we addressed this separately. We tested each of the 540 VJ combinations in our panel for interactions using logistic regression while controlling for individual donor effects. The resulting Z-scores of the interactions show a normal distribution around a mean Z-score of 0 (**Fig. C**). This strongly suggests that there is no systematic bias of the results depicted in **Fig. 1** of the main text caused by interactions of Vß and Jß genes. Of the 540 combinations tested only 19 VJ combinations (3.5%) showed significant interaction after Bonferroni correction (**Table E**).

**Figure C: Distribution of Z-scores of interaction between the 540 TCR Vß-Jß combinations.** The red line depicts a theoretical normal distribution with its mean at 0. The distribution of Z-scores follows this theoretical distribution nearly perfectly and does not deviate significantly from the normal distribution.

**Table E: Significant interactions between Vß and Jß genes**

| V-gene | J-gene | OR V-gene | OR J-gene | OR interaction | p-value |
| --- | --- | --- | --- | --- | --- |
| V29.1 | **J1.2** | 1.08 | 1.08 | 0.65 | 4.95E-40 |
| V29.1 | **J1.5** | 0.91 | 1.35 | 2.00 | 6.66E-31 |
| V29.1 | **J2.2** | 0.91 | 0.94 | 0.65 | 6.67E-28 |
| V19 | **J1.1** | 0.98 | 1.05 | 0.67 | 2.42E-27 |
| V29.1 | **J1.1** | 0.91 | 1.05 | 1.37 | 2.64E-27 |
| V29.1 | **J2.3** | 0.91 | 1.22 | 1.37 | 6.43E-20 |
| V29.1 | **J2.5** | 0.91 | 1.33 | 1.39 | 2.04E-18 |
| V6.5 | **J2.5** | 1.23 | 1.33 | 0.69 | 3.21E-10 |
| V19 | **J2.2** | 0.98 | 0.94 | 1.32 | 2.94E-07 |
| V29.1 | **J2.6** | 0.91 | 1.37 | 0.64 | 5.05E-07 |
| V19 | **J1.2** | 0.98 | 1.08 | 1.25 | 2.64E-06 |
| V6.6 | **J2.5** | 1.23 | 1.33 | 0.72 | 1.99E-05 |
| V6.5 | **J2.3** | 1.23 | 1.22 | 0.79 | 3.39E-05 |
| V6.2 | **J2.7** | 1.32 | 0.67 | 1.25 | 4.24E-05 |
| V27 | **J2.2** | 0.26 | 0.94 | 1.33 | 4.33E-05 |
| V6.5 | **J2.7** | 1.23 | 0.67 | 1.18 | 5.06E-05 |
| V12.3 | **J2.3** | 1.59 | 1.22 | 0.73 | 8.44E-05 |
| V12.3 | **J2.5** | 1.59 | 1.33 | 0.72 | 8.59E-05 |
| V12.3 | **J2.7** | 1.59 | 0.67 | 1.27 | 9.21E-05 |

**Additional results on associations of CDR3ß with CD4^+^/CD8^+^propensity: Fig.** **D** shows the extensive analysis of the main text describing **Fig. 1C**. **Fig. 1C** in the main text is depicted here as **panel E.**).

**Figure D: Analysis of biochemical properties of different parts of TCRß chain. (A)** The associations with CD4+/CD8+ propensity of the different motifs were determined by adding each motif to a null model of the dataset. Subsequently the improvement of the model’s fit to the data was calculated as the improvement (decrease) of the Akaiki Information Criterium value (note that for clarity -AIC is plotted). The motifs investigated were the Vß genes (all 42 genes combined), Jß genes (all 13 genes combined), CDR3ß net charge and number of aromatic, aliphatic or polar groups in CDR3ß. **(B)** same as (A) but now conditioned (arrow) on the Vß genes to correct for potential interactions of the motifs with the Vß genes association. After correction the observed effects in the CDR3ß and that of the Jß genes remain, indication that they are independent. **(C)** same as (A) but now conditioned on the Vß variants and the CDR3ß net charge. **(D)** same as (A) but now conditioned on the Vß variants, CDR3ß net charge, Jß genes. **(E)** Plot of CDR3ß net charge versus CD4+/CD8+ propensity. The analysis was performed per donor, so the dots show the mean of the 18 donors (error bars = 95% CI). A quasi-linear relation was observed. **(F-H)** Same as (E) but now for the presence of aromatic, polar amino and aliphatic amino acids.

**Additional results on associations within the Vß gene segment with CD4^+^/CD8^+^propensity:** As the model of charged, aromatic, polar and aliphatic groups worked so well for the CDR3s associations with CD4^+^/CD8^+^ propensity, we investigated whether this model also explained the associations found for Vß genes and Jß genes. Therefore we divided each TCRβ sequence into 6 non-overlapping regions: 5 regions encoded by Vβ genes (FR1-3 and CDR1-2), and the hypervariable CDR3 at the junction of the Vβ and Jβ segments. For each TCRß sequence we counted the number aromatic, aliphatic and polar side-chains and calculated the net charge. We performed the same stepwise regression analysis as described for the data presented in **Fig. D** and **Fig E**. First we determined for each of the variable whether addition of the variable would improve the null-model. We found that CDR3ß net charge provided the most improvement of the model (5,436 AIC units), but we also found strong improvement for other variables such as CDR1ß net charge (1,702 AIC units), the number of aromatic groups in the CDR3ß (1523 AIC units) and the CDR2ß net charge (1,241 AIC units) (**Fig. E panel A**). Stepwise regression showed that these variables were mainly independent (**Fig E panels B-F.**).

After 5 steps of regression the AIC improvement of the Vß genes (all 41 Vß genes combined) was still 9,831 AIC units and that of the Jß genes (all 13 Jß genes combined) was still 1,673 AIC units). This means that the model used can only explain part of the Vß genes and Jß genes associations with CD4^+^/CD8^+^ propensity.

**Figure E:** **Analysis of biochemical properties of different parts of TCRß chain. (A)** The associations with CD4^+^/CD8^+^ propensity of the different motifs were determined for each of the TCRß segments by adding each motif to a null model of the dataset (conditioning on interdonor variation only). Subsequently, the improvement of the model’s fit to the data was calculated as the improvement of the Akaiki Information Criterium value (AIC_motif_-AIC_null_). As reference the Vß genes (all 41 genes combined), Jß genes (all 13 genes combined) are shown in black. **(B)** same as (A) but now the null model includes the CDR3ß net charge (arrow). **(C)** same as (B), but now the null model also includes CDR1ß net charge (arrow). **(D)** same as (C), but now the null model also includes CDR2ß net charge (arrow). **(E)** same as (D), but now the null model also includes the number of aromatic groups in FR3. **(F)** same as (E), but now the null model also includes the number aliphatic groups in FR3.

**Additional results on association between CDR3β-length and CD4^+^/CD8^+^propensity .**

Similar to the net charge analysis we used stepwise regression to address associations between CDR3 length and CD4^+^ vs CD8^+^ subsets (glm function in R from stat-package version 3.0.1) **(Table F)**. In the β-chain analysis we found an association when we corrected for interdonor variation only, but this association disappeared after correction for Vβ- and Jß genes and net charge of the CDR3.

To make sure that the association of net charge was not influenced by CDR3-length we performed the reciprocal analysis **(Table G)**. We found that for the associations found for net charge in both the α and β were not influenced by CDR3 length.

**Table F: Association between CD4/CD8 subset and CDR3 length**

| chain | Output variable | Independent variable | Corrected for effects of | OR & 95%-CI  CDR3-length | p-value |
| --- | --- | --- | --- | --- | --- |
| TCR-β | CD4^+^ vs. CD8^+^ status | CDR3-length | Donor | 0.99 (0.98 - 0.99) | 3.8^-9^ |
|  | CD4^+^ vs. CD8^+^ status | CDR3-length | Donor + V-genes + J-genes | 0.99 (0.99 - 1.00) | 0.01 |
|  | CD4^+^ vs. CD8^+^ status | CDR3-length | Donor + V-genes + J-genes + CDR3-net charge | 1.00 (0.99 - 1.00) | 0.26 |

**Table G: Reciprocal approach of Table F to test whether the net charge association is influenced by CDR3 length**

| chain | Output variable | Independent variable | Corrected for effects of | OR & 95%-CI  CDR3-length | p-value |
| --- | --- | --- | --- | --- | --- |
| TCR-β | CD4^+^ vs. CD8^+^ status | CDR3-net charge | Donor | 0.76 (0.76 - 0.77) | <1^-100^ |
|  | CD4^+^ vs. CD8^+^ status | CDR3-net charge | Donor + V-genes + J-genes | 0.73 (0.73 - 0.74) | <1^-100^ |
|  | CD4^+^ vs. CD8^+^ status | CDR3-net charge | Donor + V-genes + J-genes + CDR3-length | 0.73 (0.73 - 0.74) | <1^-100^ |

**Additional NGS-results on TCRα: Table H** shows the amount of reads and unique TCRα rearrangements (clones) that were available from the TCRα sequencing.

**Table H: TCRα analysis: Number of NGS-reads and clones available for analysis**

|  | CD4 naïve | CD8 naïve |
| --- | --- | --- |
| Reads/donor* | 5874 (1,039 – 10,549) | 9825 (6,876 - 12,742) |
| Clones/donor** | 3729 (752 - 6473) | 5427 (4,217 - 7183) |
| Total clones | 19,501 | 28,572 |

* reads recovered after bioinfomatics; mean(range)

** clones detected; mean(range)

**Additional results on associations of CDR3α with CD4^+^/CD8^+^propensity:** As for the CDR3ß dataset we wanted to investigate potential associations of CDR3α motifs with the model based on the biochemical properties of the amino acid side chains. To this end we performed the same analysis for the CDR3α as described in **Fig. D** for the CDR3ß. We found similar results (**Fig. F**). The null-model improved most after addition of the Vα genes follow by comparable signals from the Jα- genes and the CDR3α net charge. After conditioning on the Vα genes, the CDR3α net charge and Jα genes the associations remained, showing their independence of the association of the Vα genes. Incorporating the CDR3α net charge into the null model modestly decreased the strength of the Jα genes association showing their partial interaction. Interestingly, next to the clear quasi-linear relation of CDR3α net charge, modest quasi-linear relations were observed for the number of aliphatic and aromatic groups. These data foremost confirm the association of CDR3ß charge with CD4^+^/CD8^+^propensity and the independence of the associations of the Vα genes, the CDR3α net charge and the Jα genes with CD4^+^/CD8^+^propensity.

**Figure F: Analysis of biochemical properties of different parts of TCRα-chain. (A)** The associations with CD4^+^/CD8^+^ propensity of the different motifs were determined by adding each motif to a null model of the dataset (correction for interdonor variation only). Subsequently the improvement of the model’s fit to the data was calculated as the improvement (decrease) of the Akaiki Information Criterium value (note that for clarity ΔAIC is plotted). The variables investigated were the Vα genes (all genes combined), Jα genes (all genes combined), CDR3α net charge and number of aromatic, aliphatic or polar groups in CDR3α. **(B)** same as (A) but now conditioned (arrow) on the Vα genes to correct for potential interactions of the motifs with the Vß genes association. After correction the observed effects in the CDR3α and that of the Jα genes remain, indication that they are independent. **(C)** same as (A) but now conditioned on the Vα variants and the CDR3α net charge. **(D)** same as (A) but now conditioned on the Vα variants, CDR3α net charge, Jα genes. **(E)** Plot of CDR3α net charge versus CD4^+^/CD8^+^ propensity. The analysis was performed per donor. The dots show the mean of the 18 donors, the error bars the 95% CI. A quasi-linear relation was observed. **(F-H)** Same as (E) but now for the presence of aromatic, aliphatic amino acids andpolar amino side-chains.

**Additional results on associations within the Vα gene segment with CD4^+^/CD8^+^propensity:** We performed the same analysis for the TCRα sequences (**Fig. G**) as for the TCRß sequences (described in **Fig. E**). For TCRα, we also found that the addition of CDR3α net charge gave the strongest improvement of the null model (591 AIC units, note that the dataset for TCRα was much small than the TCRß dataset resulting in smaller AIC values) (**Fig. G panel A**). The dominance of the CDR3α net charge association was far less than for the CDR3ß net charge, with additional strong associations in the FR2-3 and CDR2-3. These associations showed variable degrees of interaction (e.g. conditioning for the number of polar groups in FR2 increased the signal of the FR3 net charge) (**Fig. G panels B-F**). The most prominent associations were the number of polar groups in FR2, net charge in FR3, number of aromatic groups in FR3 and number of aliphatic groups in FR2. After 5 steps of regression there was still added improvement of the model when adding the Vα or Jα genes to the null model, suggesting that part of their signal was not explained by the model of biochemical properties.

**Figure G:** **Analysis of biochemical properties of different parts of TCRα chain. (A)** The associations with CD4^+^/CD8^+^ propensity of the different motifs were determined for each of the TCRα segments by adding each motif to a null model of the dataset (conditioning on interdonor variation only). Subsequently, the improvement of the model’s fit to the data was calculated as the improvement of the Akaiki Information Criterium value (AIC_motif_-AIC_null_). As reference the Vα genes, Jα genes are shown in black. **(B)** same as (A) but now the null model includes the CDR3ß net charge (arrow). **(C)** same as (B), but now the null model also includes the number of polar groups in FR2 (arrow). **(D)** same as (C), but now the null model also includes FR3 net charge (arrow). **(E)** same as (D), but now the null model also includes the number of aromatic groups in FR3 (arrow). **(F)** same as (E), but now the null model also includes the number aliphatic groups in FR2 (arrow).

**Additional results on association between CDR3α -length and CD4^+^/CD8^+^propensity.**

Similar to the net charge analysis we used stepwise regression to address associations between CDR3 length and CD4^+^ vs CD8^+^ subsets (glm function in R from stat-package version 3.0.1) **(Table I)**. In the β-chain analysis we found an association when we corrected for interdonor variation only, but this association disappeared after correction for Vβ- and Jß genes and net charge of the CDR3.

To make sure that the association of net charge was not influenced by CDR3-length we performed the reciprocal analysis **(Table J)**. We found that for the associations found for net charge in both the α and β were not influenced by CDR3 length.

**Table I: Association between CD4/CD8 subset and CDR3 length**

| chain | Output variable | Independent variable | Corrected for effects of | OR & 95%-CI  CDR3-length | p-value |
| --- | --- | --- | --- | --- | --- |
| TCR-α | CD4^+^ vs. CD8^+^ status | CDR3-length | Donor | 0.93 (0.92 - 0.94) | 5.1^-40^ |
|  | CD4^+^ vs. CD8^+^ status | CDR3-length | Donor + V-genes + J-genes | 0.96 (0.95 - 0.98) | 1.2^-8^ |
|  | CD4^+^ vs. CD8^+^ status | CDR3-length | Donor + V-genes + J-genes + CDR3-net charge | 0.97 (0.95 – 0.98) | 5.2^-7^ |

**Table J: Reciprocal approach of Table I to test whether the net charge association is influenced by CDR3 length**

| chain | Output variable | Independent variable | Corrected for effects of | OR & 95%-CI  CDR3-length | p-value |
| --- | --- | --- | --- | --- | --- |
| TCR-α | CD4^+^ vs. CD8^+^ status | CDR3-net charge | Donor | 0.79 (0.77 - 0.80) | <1^-100^ |
|  | CD4^+^ vs. CD8^+^ status | CDR3-net charge | Donor + V-genes + J-genes | 0.77 (0.75 - 0.79) | 1.5^-83^ |
|  | CD4^+^ vs. CD8^+^ status | CDR3-net charge | Donor + V-genes + J-genes + CDR3-length | 0.77 (0.75 - 0.79) | 5.2^-82^ |

**Table K: Variation between donors for analysis shown in Table 1 of main text.** The regression model was performed per donor to visualize the interdonor-variation.

| TCR-chain | variable | Median | IQR - Range |
| --- | --- | --- | --- |
| Beta (n=18 | V.genes | 11.2% | 9.7% - 14.9% |
| donors) | J.genes | 2.6% | 2.0% - 2.9% |
|  | CDR3 charge | 3.2% | 1.5% - 4.9% |
|  | CDR3 length | 0.1% | 0.0% - 0.4% |
|  |  |  |  |
| Alpha (n=5 | V.genes | 12.0% | 8.1% - 13.0% |
| donors) | J.genes | 3.6% | 3.3% - 3.7% |
|  | CDR3 charge | 2.2% | 0.6% - 3.0% |
|  | CDR3 length | 0.5% | 0.3% - 0.8% |
