# Supplementary material for: Somatic Variation of T-Cell Receptor Genes Strongly Associate with HLA Class Restriction
Source: PLoS One. 2015 Oct 30;10(10):e0140815. doi: 10.1371/journal.pone.0140815 (PMC4627806; doi:10.1371/journal.pone.0140815)

Propensity for Vbeta13

donor	V13	total TCRs	OR	CI	p value
1	144	13593	0.3	0.2−0.46	1e−08
2	99	12567	0.16	0.09−0.28	1e−10
3	175	14965	0.12	0.08−0.18	3e−27
4	113	12387	0.24	0.15−0.39	6e−09
5	62	8050	0.13	0.06−0.27	5e−08
6	129	12302	0.08	0.04−0.14	5e−15
7	77	9929	0.36	0.21−0.61	1e−04
8	102	12477	0.16	0.09−0.28	2e−10
9	98	13909	0.22	0.13−0.38	2e−08
10	84	13074	0.28	0.17−0.46	6e−07
11	73	12150	0.14	0.08−0.28	4e−09
12	44	13082	0.34	0.18−0.65	0.001
13	145	14911	0.28	0.19−0.4	3e−11
14	46	11142	0.51	0.28−0.94	0.03
15	103	10303	0.42	0.27−0.64	5e−05
16	102	11252	0.17	0.1−0.29	7e−11
17	50	8005	0.02	0−0.16	2e−04
18	50	7151	0.14	0.08−0.25	6e−11
**random effects model**	**1696**	**211249**	**0.21**	**0.16−0.27**	**5e−37**
**fixed effects model**	**1696**	**211249**	**0.22**	**0.19−0.25**	**<1e−100**

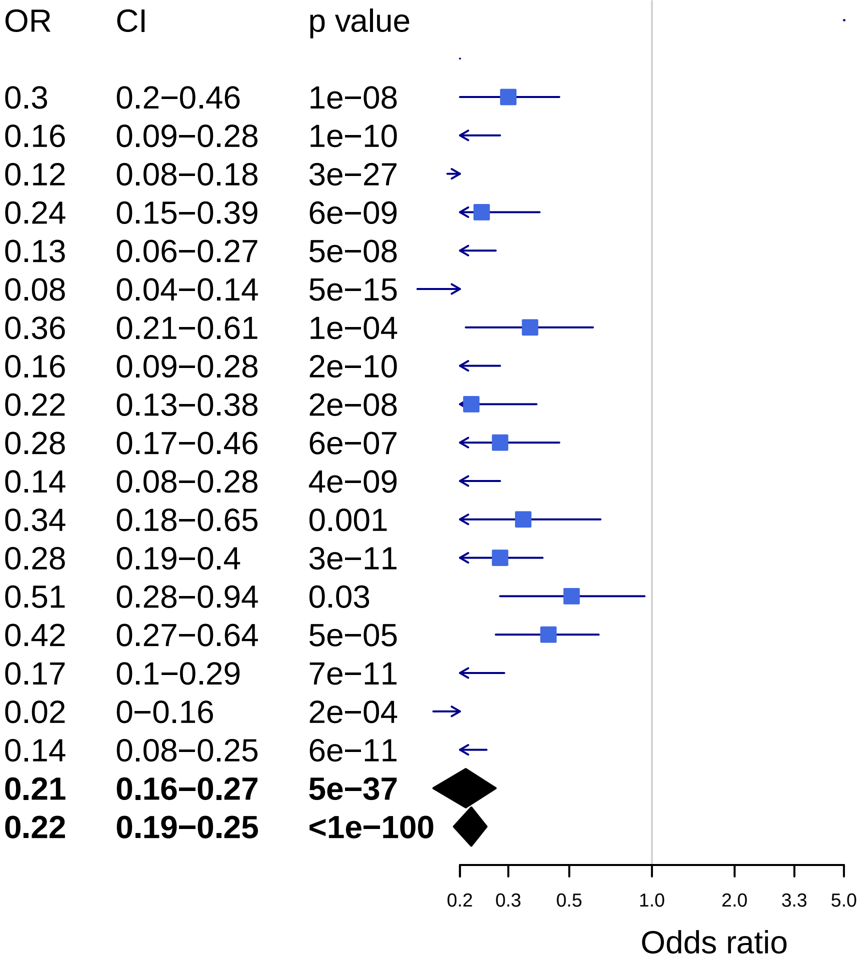

Propensity for Vbeta27

donor	V27	total TCRs	OR	CI	p value
1	738	13593	0.23	0.19−0.28	1e−49
2	1548	12567	0.17	0.15−0.19	<1e−100
3	492	14965	0.27	0.22−0.32	6e−42
4	1675	12387	0.22	0.2−0.25	<1e−100
5	698	8050	0.31	0.26−0.37	4e−39
6	1105	12302	0.35	0.31−0.4	3e−52
7	885	9929	0.21	0.18−0.25	2e−68
8	1421	12477	0.25	0.22−0.29	7e−96
9	1352	13909	0.33	0.29−0.38	6e−64
10	1283	13074	0.26	0.23−0.3	3e−89
11	899	12150	0.18	0.15−0.21	4e−92
12	1069	13082	0.21	0.18−0.24	1e−99
13	1383	14911	0.25	0.22−0.29	<1e−100
14	288	11142	0.37	0.29−0.48	2e−14
15	584	10303	0.33	0.27−0.4	2e−31
16	1060	11252	0.18	0.16−0.22	7e−99
17	524	8005	0.1	0.07−0.14	2e−45
18	333	7151	0.16	0.13−0.2	5e−55
**random effects model**	**17337**	**211249**	**0.24**	**0.21−0.27**	**<1e−100**
**fixed effects model**	**17337**	**211249**	**0.24**	**0.23−0.25**	**<1e−100**

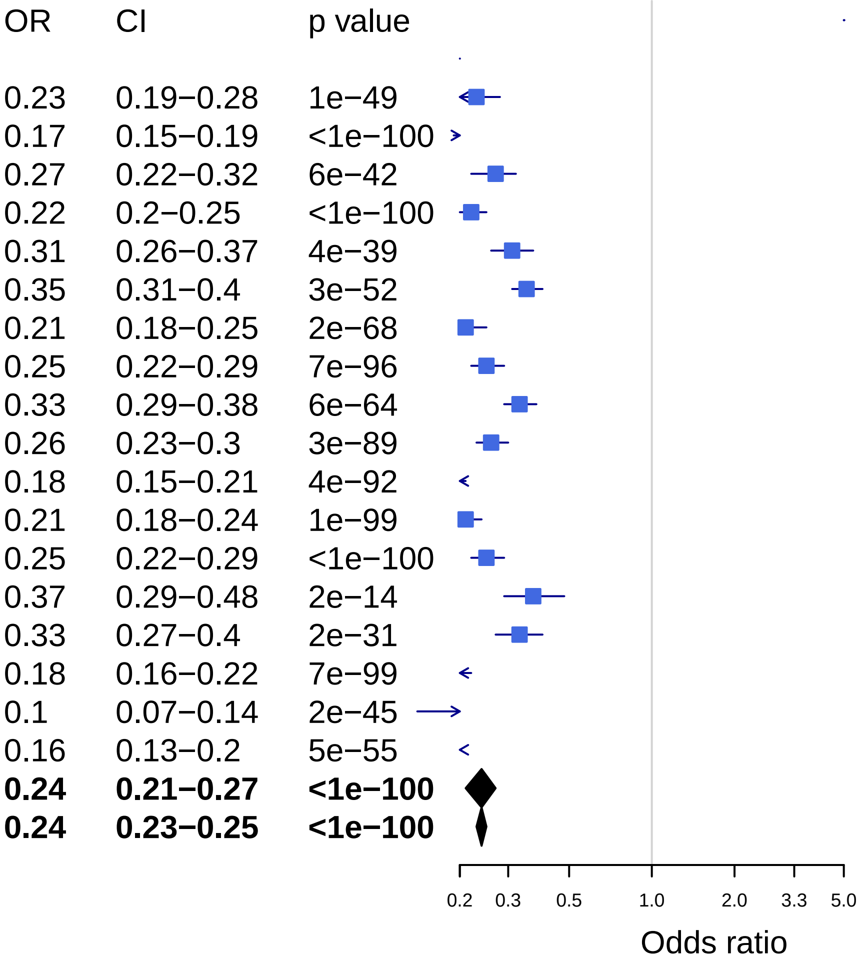

Propensity for Vbeta7.6

donor	V7.6	total TCRs	OR	CI	p value
1	36	13593	0.54	0.26−1.11	0.09
2	16	12567	0.98	0.36−2.62	1
3	38	14965	0.23	0.11−0.46	4e−05
4	39	12387	0.3	0.14−0.64	0.002
5	15	8050	0.65	0.23−1.84	0.4
6	36	12302	0.18	0.07−0.43	1e−04
7	35	9929	0.82	0.41−1.61	0.6
8	43	12477	0.62	0.33−1.16	0.1
9	38	13909	0.24	0.1−0.54	6e−04
10	43	13074	0.42	0.22−0.8	0.009
11	25	12150	0.75	0.34−1.66	0.5
12	13	13082	0.48	0.16−1.5	0.2
13	37	14911	0.45	0.23−0.89	0.02
14	17	11142	3.86	1.1−13.59	0.04
15	20	10303	0.8	0.33−1.93	0.6
16	23	11252	0.36	0.15−0.88	0.03
17	50	8005	0	0−1.32613144797118e+98	0.9
18	24	7151	0.47	0.2−1.09	0.08
**random effects model**	**548**	**211249**	**0.5**	**0.38−0.65**	**7e−07**
**fixed effects model**	**548**	**211249**	**0.48**	**0.4−0.59**	**2e−13**

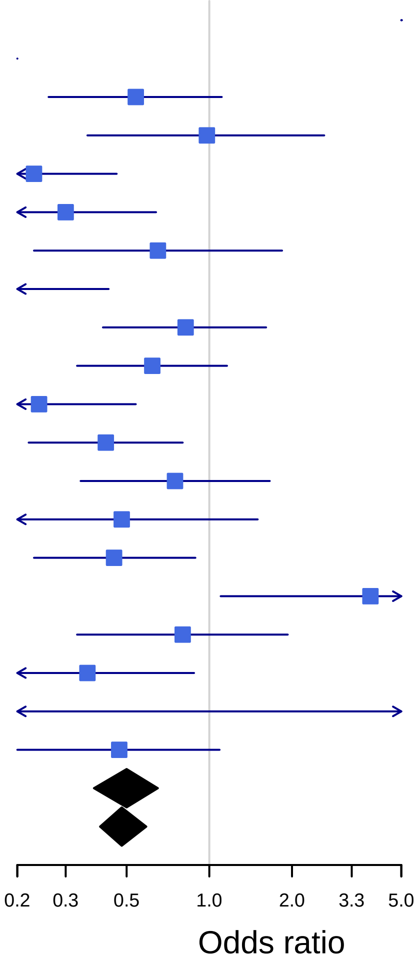

Propensity for Vbeta7.9

donor	V7.9	total TCRs	OR	CI	p value
1	125	13593	0.47	0.31−0.7	2e−04
2	102	12567	0.64	0.43−0.95	0.03
3	99	14965	0.24	0.16−0.37	6e−11
4	96	12387	0.45	0.29−0.7	3e−04
5	88	8050	0.52	0.34−0.81	0.004
6	107	12302	0.31	0.2−0.48	2e−07
7	35	9929	0.61	0.31−1.23	0.2
8	245	12477	0.43	0.33−0.57	2e−09
9	112	13909	0.39	0.26−0.6	2e−05
10	73	13074	0.83	0.52−1.33	0.4
11	24	12150	0.82	0.36−1.83	0.6
12	29	13082	0.69	0.33−1.45	0.3
13	58	14911	0.44	0.25−0.76	0.003
14	21	11142	2.66	0.97−7.34	0.06
15	32	10303	0.76	0.37−1.53	0.4
16	31	11252	0.47	0.23−1	0.05
17	64	8005	0.04	0.01−0.16	7e−06
18	25	7151	0.47	0.2−1.07	0.07
**random effects model**	**1366**	**211249**	**0.49**	**0.4−0.61**	**2e−10**
**fixed effects model**	**1366**	**211249**	**0.47**	**0.42−0.53**	**3e−36**

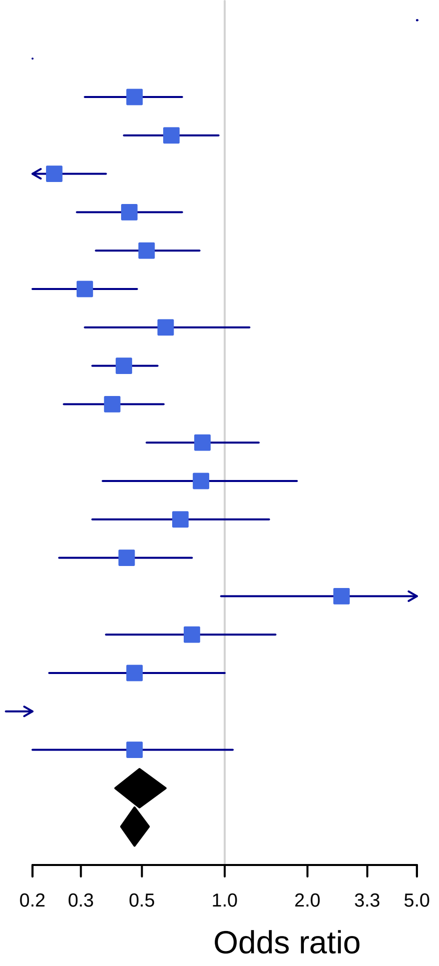

Propensity for Vbeta7.4

donor	V7.4	total TCRs	OR	CI	p value
1	5	13593	0	0−4.72551542944282e+69	0.9
2	1	12567	0	0−1.07117322877729e+97	0.9
3	7	14965	1.35	0.26−7.08	0.7
4	7	12387	0.81	0.18−3.68	0.8
5	1	8050	0	0−1.053135666789e+97	0.9
6	8	12302	0.24	0.05−1.22	0.09
7	4	9929	1.31	0.18−9.41	0.8
8	4	12477	2.3	0.23−22.7	0.5
9	6	13909	1.06	0.21−5.34	0.9
10	8	13074	0.52	0.12−2.2	0.4
11	2	12150	0	0−2.33099364651526e+113	0.9
12	5	13082	1.18	0.2−7.15	0.9
13	3	14911	1.14	0.1−12.73	0.9
14	1	11142	0	0−1.4330438077742e+97	0.9
15	3	10303	0	0−4.95496120985884e+89	0.9
16	5	11252	0	0−1.61507652514923e+116	0.9
17	4	8005	0	0−3.9441208559128e+131	0.9
18	5	7151	0.4	0.06−2.44	0.3
**random effects model**	**79**	**211249**	**0.78**	**0.45−1.36**	**0.4**
**fixed effects model**	**79**	**211249**	**0.78**	**0.45−1.36**	**0.4**

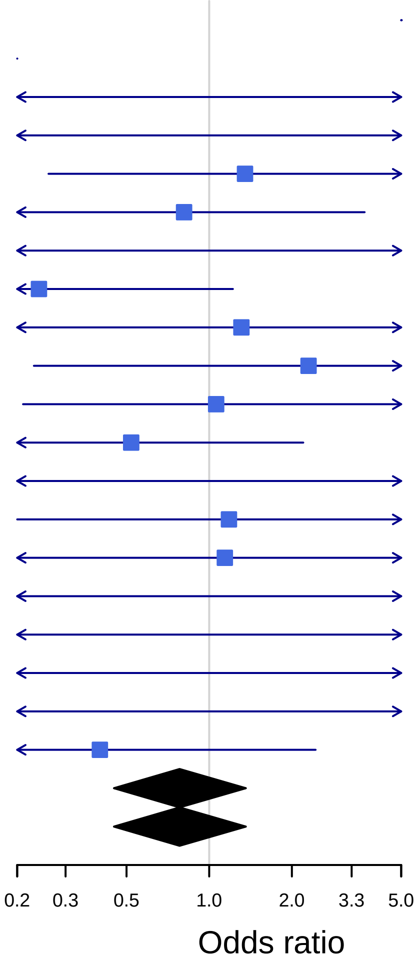

Propensity for Vbeta4.1

donor	V4.1	total TCRs	OR	CI	p value
1	105	13593	0.3	0.18−0.48	9e−07
2	138	12567	0.74	0.52−1.04	0.08
3	63	14965	0.48	0.29−0.8	0.004
4	212	12387	0.44	0.33−0.59	5e−08
5	38	8050	0.34	0.17−0.69	0.003
6	105	12302	0.51	0.34−0.76	0.001
7	93	9929	0.6	0.39−0.93	0.02
8	126	12477	0.3	0.2−0.45	8e−09
9	138	13909	0.49	0.34−0.71	1e−04
10	75	13074	0.62	0.39−0.98	0.04
11	44	12150	0.98	0.53−1.79	0.9
12	28	13082	0.37	0.17−0.83	0.02
13	104	14911	0.57	0.39−0.85	0.006
14	5	11142	2.72	0.29−25.14	0.4
15	99	10303	0.42	0.27−0.65	8e−05
16	72	11252	0.66	0.41−1.06	0.09
17	39	8005	0	0−7.75304008210213e+111	0.9
18	21	7151	0.15	0.06−0.37	3e−05
**random effects model**	**1505**	**211249**	**0.49**	**0.41−0.58**	**2e−16**
**fixed effects model**	**1505**	**211249**	**0.49**	**0.44−0.55**	**7e−36**

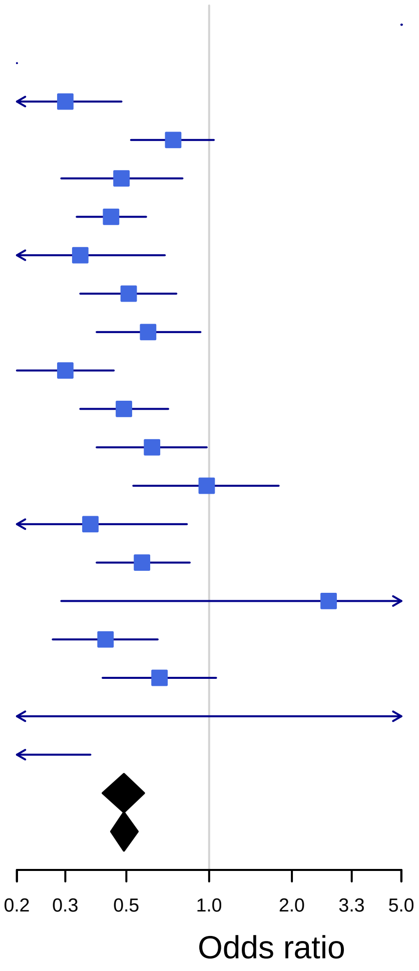

Propensity for Vbeta7.8

donor	V7.8	total TCRs	OR	CI	p value
1	359	13593	0.59	0.47−0.74	4e−06
2	166	12567	0.74	0.54−1	0.05
3	205	14965	0.54	0.41−0.71	1e−05
4	269	12387	0.44	0.34−0.58	2e−09
5	146	8050	0.46	0.33−0.66	2e−05
6	226	12302	0.4	0.3−0.53	2e−10
7	154	9929	0.81	0.58−1.12	0.2
8	251	12477	0.57	0.44−0.74	2e−05
9	248	13909	0.66	0.51−0.85	0.002
10	201	13074	0.75	0.56−0.99	0.04
11	135	12150	0.46	0.32−0.66	2e−05
12	139	13082	0.75	0.53−1.05	0.09
13	246	14911	0.86	0.66−1.1	0.2
14	124	11142	0.92	0.64−1.31	0.6
15	148	10303	0.26	0.17−0.38	1e−11
16	139	11252	0.64	0.46−0.91	0.01
17	76	8005	0.19	0.1−0.36	5e−07
18	120	7151	0.14	0.1−0.21	4e−24
**random effects model**	**3352**	**211249**	**0.52**	**0.43−0.64**	**7e−11**
**fixed effects model**	**3352**	**211249**	**0.56**	**0.52−0.6**	**4e−57**

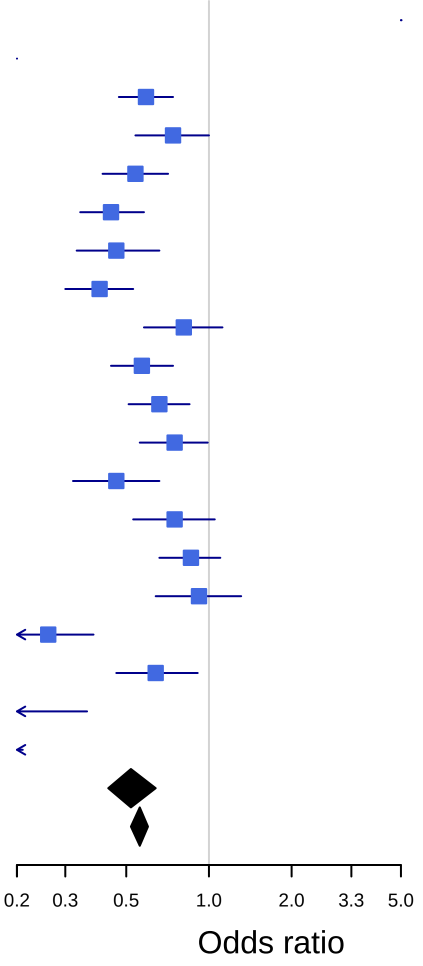

Propensity for Vbeta5.6

donor	V5.6	total TCRs	OR	CI	p value
1	354	13593	0.64	0.51−0.8	7e−05
2	261	12567	0.52	0.4−0.67	4e−07
3	314	14965	0.49	0.39−0.61	4e−10
4	283	12387	0.66	0.52−0.84	9e−04
5	211	8050	0.33	0.25−0.46	3e−12
6	332	12302	0.31	0.24−0.4	3e−20
7	227	9929	0.34	0.25−0.47	6e−12
8	213	12477	0.69	0.53−0.92	0.01
9	358	13909	0.65	0.52−0.81	1e−04
10	243	13074	0.67	0.52−0.87	0.003
11	212	12150	0.39	0.29−0.51	1e−10
12	164	13082	1.05	0.76−1.44	0.8
13	305	14911	0.93	0.74−1.17	0.5
14	185	11142	0.49	0.36−0.67	5e−06
15	208	10303	0.62	0.47−0.82	8e−04
16	212	11252	0.57	0.43−0.75	8e−05
17	139	8005	0.62	0.43−0.89	0.01
18	94	7151	0.7	0.44−1.11	0.1
**random effects model**	**4315**	**211249**	**0.56**	**0.48−0.66**	**2e−13**
**fixed effects model**	**4315**	**211249**	**0.57**	**0.53−0.6**	**2e−68**

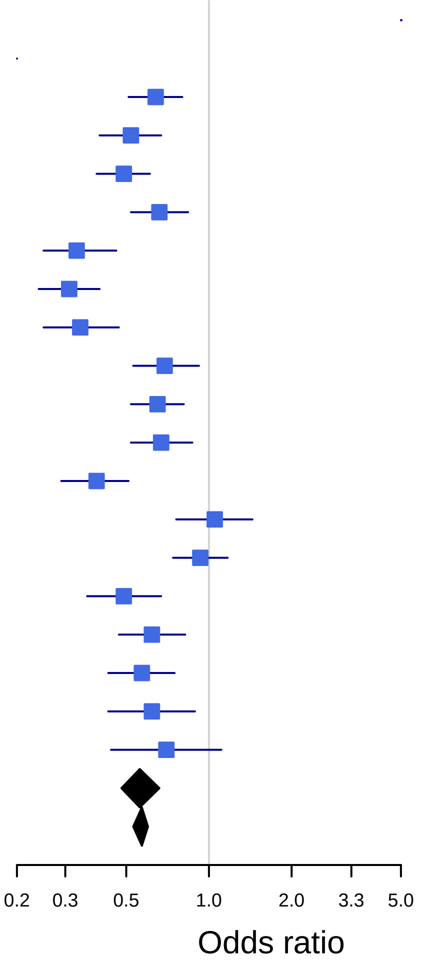

Propensity for Vbeta12.5

donor	V12.5	total TCRs	OR	CI	p value
1	110	13593	0.49	0.32−0.74	7e−04
2	64	12567	0.83	0.5−1.35	0.4
3	112	14965	1.35	0.88−2.06	0.2
4	39	12387	0.96	0.51−1.8	0.9
5	48	8050	0.31	0.17−0.6	4e−04
6	43	12302	0.53	0.29−0.99	0.05
7	53	9929	1.47	0.85−2.56	0.2
8	53	12477	0.65	0.37−1.14	0.1
9	141	13909	0.56	0.4−0.8	0.001
10	51	13074	0.92	0.53−1.6	0.8
11	31	12150	0.44	0.21−0.92	0.03
12	35	13082	0.61	0.31−1.19	0.1
13	107	14911	0.73	0.5−1.07	0.1
14	33	11142	1.16	0.57−2.36	0.7
15	56	10303	0.54	0.31−0.93	0.03
16	32	11252	0.32	0.15−0.7	0.004
17	20	8005	0.42	0.15−1.17	0.1
18	7	7151	0.19	0.04−0.87	0.03
**random effects model**	**1035**	**211249**	**0.66**	**0.54−0.82**	**1e−04**
**fixed effects model**	**1035**	**211249**	**0.68**	**0.6−0.78**	**7e−09**

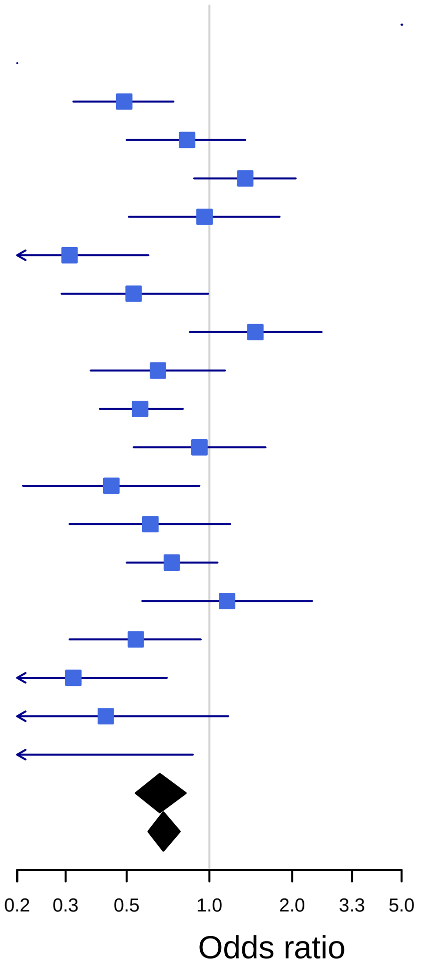

Propensity for Vbeta14

donor	V14	total TCRs	OR	CI	p value
1	139	13593	0.74	0.52−1.05	0.09
2	92	12567	1.31	0.86−1.98	0.2
3	122	14965	0.28	0.19−0.41	4e−11
4	44	12387	0.93	0.51−1.7	0.8
5	50	8050	0.81	0.46−1.42	0.5
6	36	12302	0.74	0.38−1.45	0.4
7	66	9929	0.55	0.33−0.93	0.02
8	80	12477	0.82	0.52−1.28	0.4
9	86	13909	0.6	0.39−0.94	0.02
10	110	13074	0.87	0.59−1.27	0.5
11	44	12150	0.96	0.53−1.74	0.9
12	52	13082	1.07	0.62−1.87	0.8
13	95	14911	0.74	0.49−1.11	0.1
14	33	11142	5.81	2.03−16.63	0.001
15	46	10303	0.84	0.47−1.52	0.6
16	45	11252	1.14	0.63−2.07	0.7
17	66	8005	0.12	0.05−0.28	8e−07
18	44	7151	0.59	0.31−1.14	0.1
**random effects model**	**1250**	**211249**	**0.76**	**0.59−0.97**	**0.03**
**fixed effects model**	**1250**	**211249**	**0.73**	**0.65−0.82**	**2e−07**

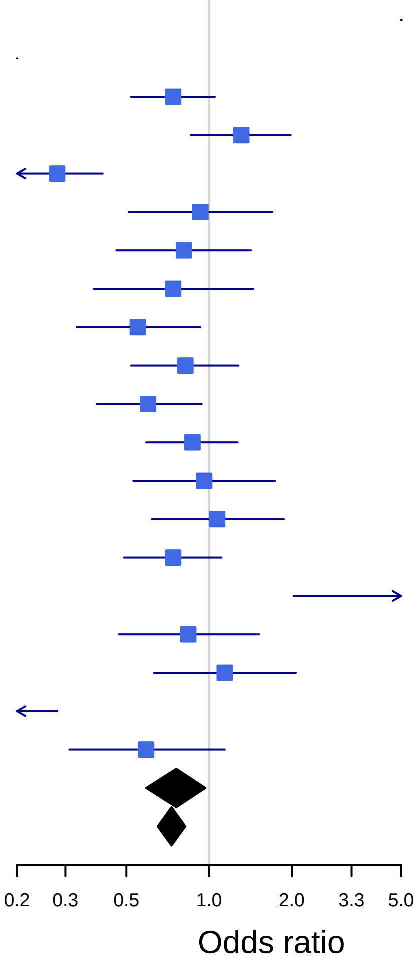

Propensity for Vbeta11.2

donor	V11.2	total TCRs	OR	CI	p value
1	496	13593	1.02	0.85−1.22	0.9
2	178	12567	0.58	0.42−0.78	4e−04
3	406	14965	0.47	0.38−0.57	9e−14
4	399	12387	0.83	0.68−1.02	0.07
5	166	8050	0.79	0.58−1.08	0.1
6	429	12302	0.73	0.6−0.88	0.001
7	309	9929	0.37	0.29−0.48	6e−14
8	171	12477	0.88	0.65−1.2	0.4
9	665	13909	0.5	0.42−0.59	2e−16
10	411	13074	1.01	0.83−1.23	0.9
11	120	12150	0.84	0.59−1.21	0.4
12	179	13082	0.83	0.62−1.12	0.2
13	271	14911	0.82	0.65−1.05	0.1
14	204	11142	4.67	3.19−6.84	2e−15
15	372	10303	0.96	0.77−1.18	0.7
16	226	11252	0.7	0.54−0.91	0.009
17	124	8005	0.12	0.06−0.22	2e−11
18	114	7151	0.4	0.27−0.58	3e−06
**random effects model**	**5240**	**211249**	**0.72**	**0.58−0.9**	**0.003**
**fixed effects model**	**5240**	**211249**	**0.73**	**0.69−0.77**	**2e−26**

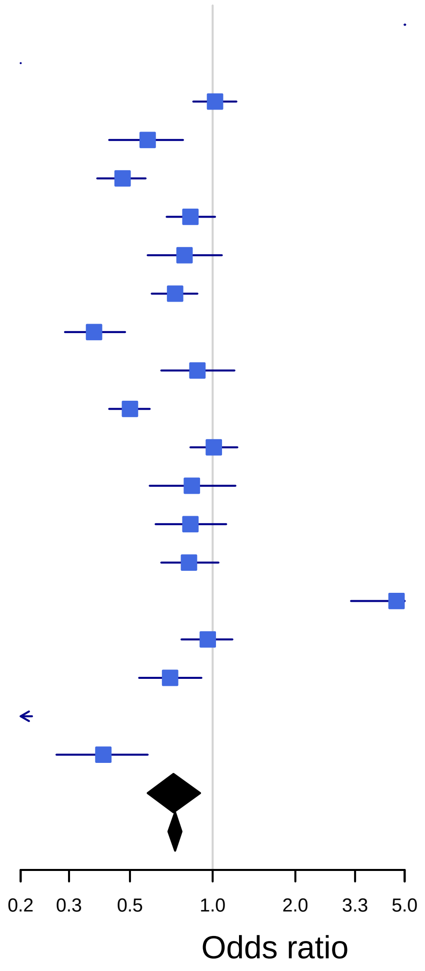

Propensity for Vbeta15

donor	V15	total TCRs	OR	CI	p value
1	259	13593	0.85	0.66−1.1	0.2
2	290	12567	0.65	0.51−0.82	4e−04
3	296	14965	1.51	1.16−1.98	0.002
4	282	12387	0.91	0.72−1.16	0.5
5	199	8050	0.55	0.41−0.74	6e−05
6	244	12302	0.91	0.7−1.17	0.5
7	219	9929	0.96	0.73−1.26	0.8
8	242	12477	0.81	0.63−1.05	0.1
9	384	13909	1.05	0.85−1.28	0.7
10	258	13074	1.5	1.16−1.95	0.002
11	225	12150	0.65	0.5−0.85	0.002
12	297	13082	0.73	0.58−0.93	0.009
13	470	14911	0.82	0.68−0.99	0.04
14	190	11142	0.52	0.39−0.7	2e−05
15	189	10303	0.98	0.73−1.31	0.9
16	271	11252	0.79	0.62−1.01	0.06
17	130	8005	1.07	0.76−1.53	0.7
18	125	7151	0.67	0.45−1	0.05
**random effects model**	**4570**	**211249**	**0.85**	**0.75−0.97**	**0.02**
**fixed effects model**	**4570**	**211249**	**0.86**	**0.81−0.91**	**7e−07**

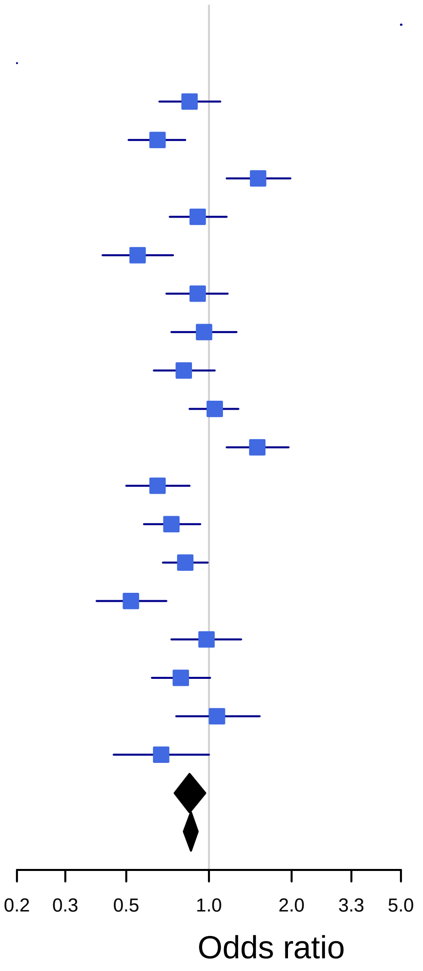

Propensity for Vbeta5.8

donor	V5.8	total TCRs	OR	CI	p value
1	8	13593	1.08	0.27−4.41	0.9
2	6	12567	1.87	0.34−10.33	0.5
3	8	14965	0.21	0.04−1.05	0.06
4	10	12387	1.46	0.4−5.26	0.6
5	5	8050	0.69	0.11−4.21	0.7
6	5	12302	3.87	0.43−35.07	0.2
7	5	9929	1.85	0.31−11.16	0.5
8	6	12477	0.16	0.02−1.36	0.09
9	11	13909	0.91	0.27−2.99	0.9
10	7	13074	0.17	0.02−1.44	0.1
11	5	12150	1.01	0.17−6.1	1
12	7	13082	1.96	0.38−10.22	0.4
13	6	14911	90800.92	0−4.43643764723263e+72	0.9
14	5	11142	3.32	0.36−30.55	0.3
15	3	10303	0.34	0.03−3.94	0.4
16	7	11252	1.95	0.37−10.24	0.4
17	8	8005	0.17	0.02−1.41	0.1
18	4	7151	0.09	0.01−0.91	0.04
**random effects model**	**116**	**211249**	**0.83**	**0.51−1.34**	**0.4**
**fixed effects model**	**116**	**211249**	**0.85**	**0.56−1.3**	**0.4**

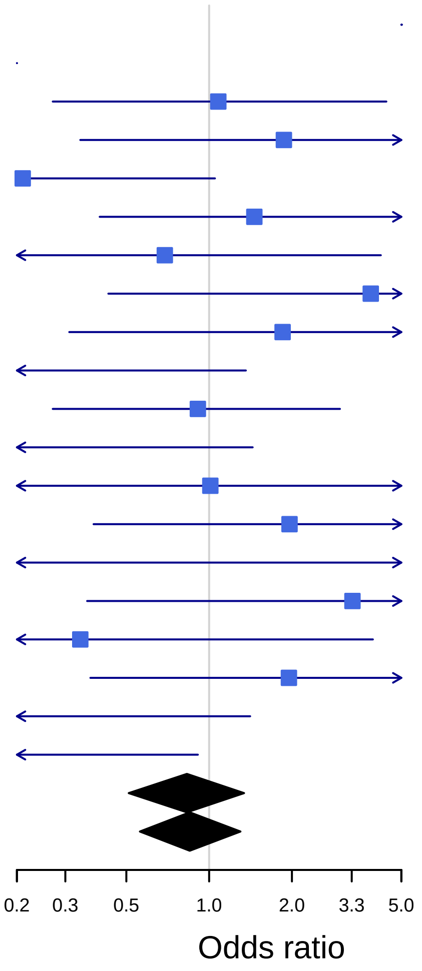

Propensity for Vbeta10.2

donor	V10.2	total TCRs	OR	CI	p value
1	5	13593	1.6	0.26−9.65	0.6
2	5	12567	0.25	0.03−2.26	0.2
3	12	14965	2.68	0.58−12.38	0.2
4	7	12387	2.4	0.46−12.57	0.3
5	3	8050	1.95	0.17−21.96	0.6
6	21	12302	1.25	0.52−2.97	0.6
7	14	9929	0.2	0.04−0.89	0.03
8	5	12477	1.16	0.19−7.19	0.9
9	22	13909	0.87	0.37−2.03	0.8
10	8	13074	0.8	0.2−3.21	0.7
11	4	12150	0.7	0.1−5.04	0.7
12	17	13082	1.31	0.48−3.59	0.6
13	13	14911	0.91	0.3−2.74	0.9
14	1	11142	28738.1	0−1.41813642557389e+106	0.9
15	9	10303	0.75	0.2−2.86	0.7
16	11	11252	1.05	0.32−3.48	0.9
17	8	8005	0	0−2.14629234227456e+91	0.9
18	1	7151	0	0−1.32300187312725e+162	0.9
**random effects model**	**166**	**211249**	**1**	**0.71−1.4**	**1**
**fixed effects model**	**166**	**211249**	**1**	**0.71−1.4**	**1**

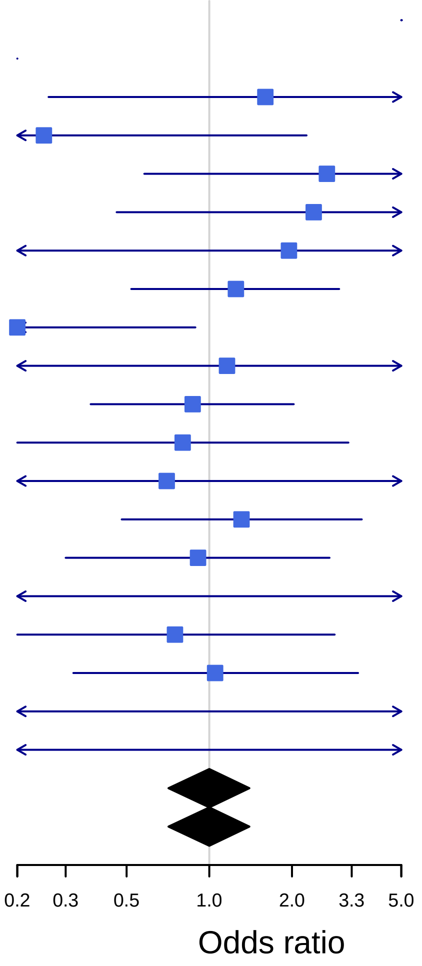

Propensity for Vbeta5.7

donor	V5.7	total TCRs	OR	CI	p value
1	18	13593	0.77	0.29−1.99	0.6
2	10	12567	2.11	0.54−8.22	0.3
3	77	14965	9.65	3.52−26.48	1e−05
4	17	12387	0.55	0.2−1.51	0.3
5	9	8050	1.05	0.28−3.95	0.9
6	25	12302	0.47	0.21−1.07	0.07
7	205	9929	0.79	0.59−1.05	0.1
8	9	12477	1.41	0.38−5.3	0.6
9	20	13909	0.6	0.24−1.51	0.3
10	226	13074	0.96	0.74−1.25	0.8
11	188	12150	0.98	0.73−1.32	0.9
12	93	13082	1.21	0.79−1.85	0.4
13	240	14911	0.93	0.72−1.2	0.6
14	172	11142	0.68	0.5−0.92	0.01
15	208	10303	0.54	0.41−0.72	2e−05
16	221	11252	1.07	0.82−1.4	0.6
17	158	8005	0.8	0.58−1.11	0.2
18	129	7151	0.69	0.46−1.04	0.08
**random effects model**	**2025**	**211249**	**0.88**	**0.74−1.05**	**0.2**
**fixed effects model**	**2025**	**211249**	**0.85**	**0.78−0.94**	**8e−04**

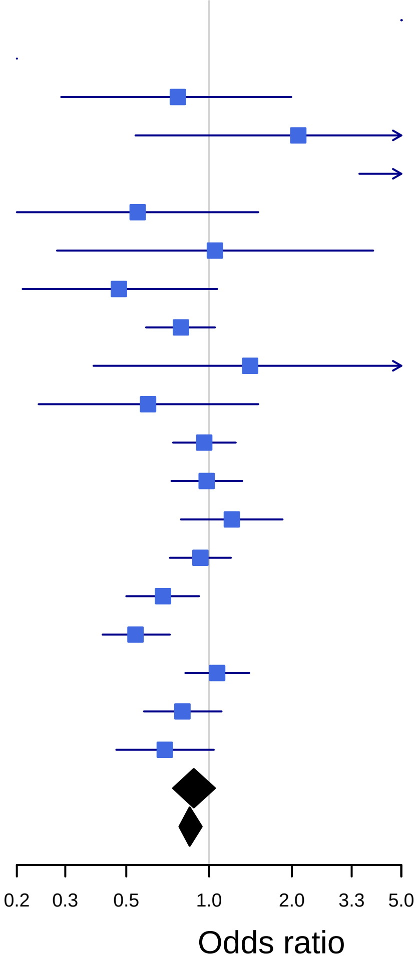

Propensity for Vbeta4.2

donor	V4.2	total TCRs	OR	CI	p value
1	101	13593	0.48	0.31−0.75	0.001
2	70	12567	0.64	0.39−1.03	0.07
3	113	14965	0.77	0.53−1.13	0.2
4	97	12387	1.59	1.06−2.4	0.03
5	112	8050	0.62	0.42−0.91	0.01
6	204	12302	0.91	0.69−1.2	0.5
7	121	9929	0.56	0.38−0.81	0.003
8	204	12477	0.78	0.59−1.03	0.08
9	93	13909	1.23	0.81−1.85	0.3
10	99	13074	1.76	1.15−2.69	0.009
11	24	12150	0.54	0.24−1.22	0.1
12	57	13082	1.4	0.8−2.45	0.2
13	119	14911	0.97	0.67−1.4	0.9
14	39	11142	3.82	1.67−8.74	0.001
15	53	10303	1.95	1.07−3.54	0.03
16	73	11252	2.22	1.33−3.7	0.002
17	26	8005	0.05	0.01−0.37	0.003
18	43	7151	0.48	0.26−0.91	0.02
**random effects model**	**1648**	**211249**	**0.95**	**0.75−1.21**	**0.7**
**fixed effects model**	**1648**	**211249**	**0.92**	**0.83−1.02**	**0.1**

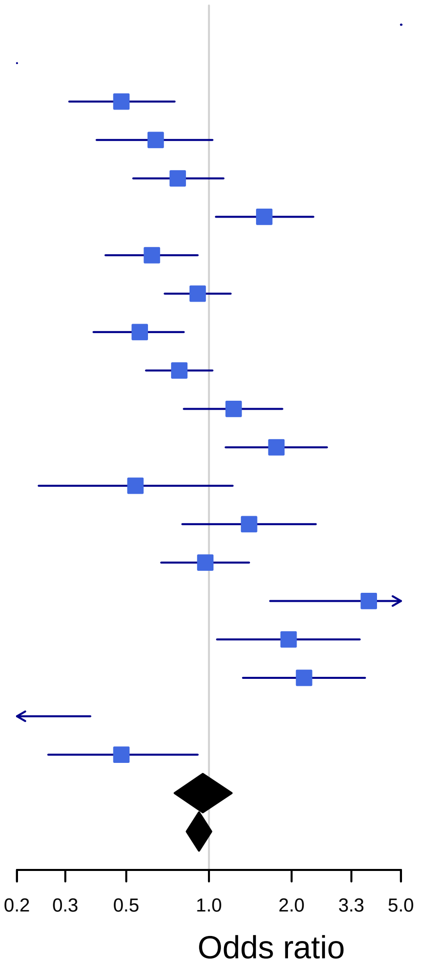

Propensity for Vbeta29.1

donor	V29.1	total TCRs	OR	CI	p value
1	3180	13593	0.81	0.75−0.88	4e−07
2	2755	12567	0.97	0.89−1.06	0.5
3	2319	14965	0.94	0.86−1.04	0.2
4	2009	12387	1.22	1.11−1.34	6e−05
5	1432	8050	0.75	0.67−0.84	1e−06
6	1503	12302	0.67	0.6−0.75	9e−13
7	1871	9929	1.25	1.13−1.39	2e−05
8	2141	12477	0.99	0.9−1.09	0.8
9	2599	13909	0.88	0.81−0.96	0.005
10	3063	13074	0.89	0.82−0.97	0.006
11	4612	12150	1.41	1.31−1.52	7e−19
12	4926	13082	0.82	0.76−0.88	1e−07
13	2575	14911	0.7	0.64−0.76	2e−16
14	4714	11142	0.37	0.34−0.4	<1e−100
15	2169	10303	0.81	0.74−0.89	2e−05
16	2973	11252	0.81	0.74−0.88	1e−06
17	2910	8005	2.44	2.22−2.68	7e−75
18	2105	7151	3.45	2.93−4.06	2e−49
**random effects model**	**49856**	**211249**	**0.98**	**0.8−1.2**	**0.9**
**fixed effects model**	**49856**	**211249**	**0.92**	**0.9−0.94**	**4e−14**

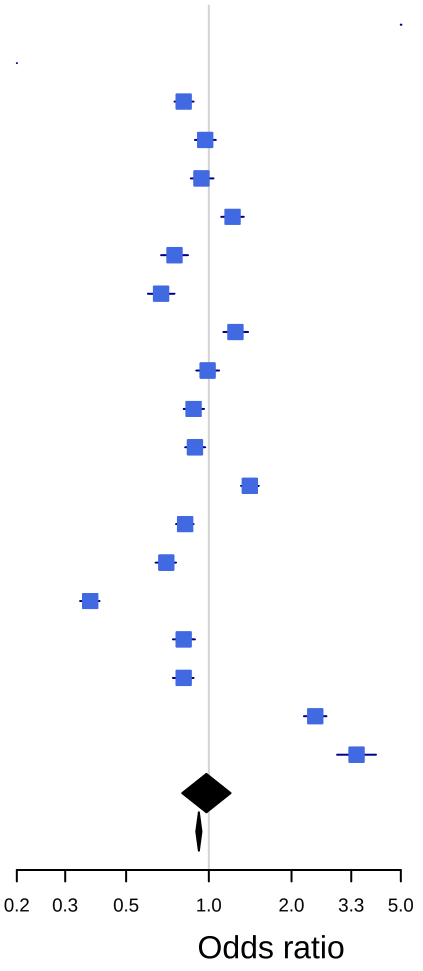

Propensity for Vbeta19

donor	V19	total TCRs	OR	CI	p value
1	1400	13593	1.27	1.14−1.42	3e−05
2	1306	12567	1.47	1.3−1.65	2e−10
3	2132	14965	0.77	0.7−0.85	7e−08
4	1245	12387	1.12	1−1.26	0.06
5	820	8050	0.82	0.71−0.95	0.007
6	1406	12302	0.76	0.68−0.85	2e−06
7	840	9929	1.19	1.03−1.38	0.02
8	1324	12477	1.2	1.07−1.35	0.002
9	1552	13909	0.84	0.76−0.94	0.002
10	1437	13074	0.52	0.46−0.58	1e−29
11	1095	12150	0.86	0.76−0.98	0.02
12	1382	13082	1.26	1.12−1.42	9e−05
13	1683	14911	1.08	0.97−1.2	0.2
14	1163	11142	1.01	0.89−1.14	0.9
15	1556	10303	0.65	0.58−0.73	3e−14
16	1004	11252	1.02	0.89−1.16	0.8
17	726	8005	0.74	0.63−0.87	2e−04
18	1027	7151	1.45	1.2−1.74	8e−05
**random effects model**	**23098**	**211249**	**0.96**	**0.84−1.1**	**0.6**
**fixed effects model**	**23098**	**211249**	**0.95**	**0.92−0.97**	**1e−04**

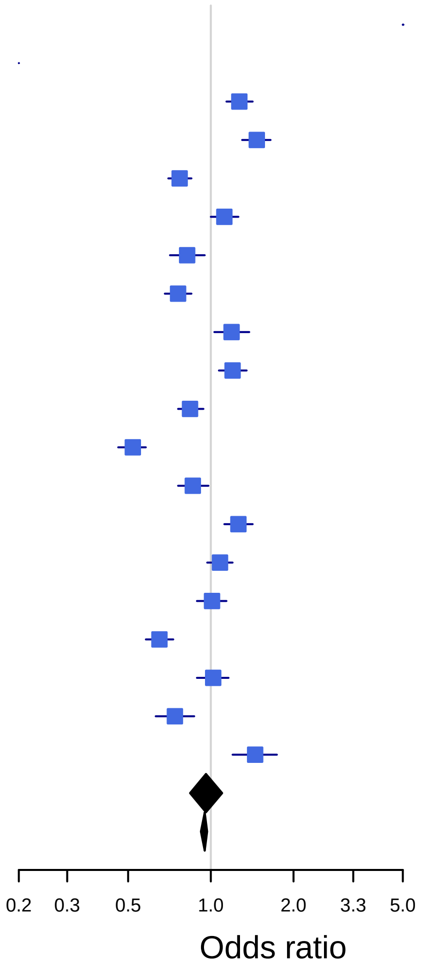

Propensity for Vbeta6.4

donor	V6.4	total TCRs	OR	CI	p value
1	28	13593	1.27	0.6−2.68	0.5
2	23	12567	1.61	0.7−3.74	0.3
3	30	14965	1.21	0.55−2.66	0.6
4	32	12387	1.11	0.55−2.24	0.8
5	17	8050	0.72	0.28−1.89	0.5
6	23	12302	1.36	0.58−3.16	0.5
7	18	9929	1.79	0.69−4.65	0.2
8	21	12477	1.27	0.53−3.06	0.6
9	24	13909	0.9	0.4−2.03	0.8
10	13	13074	258158.58	0−1.73316717105453e+81	0.9
11	20	12150	0.64	0.26−1.56	0.3
12	31	13082	0.79	0.38−1.6	0.5
13	29	14911	1.15	0.55−2.42	0.7
14	25	11142	2.13	0.88−5.17	0.09
15	23	10303	0.74	0.32−1.71	0.5
16	17	11252	0.83	0.32−2.16	0.7
17	14	8005	0.2	0.05−0.92	0.04
18	33	7151	0.14	0.07−0.3	1e−07
**random effects model**	**421**	**211249**	**0.91**	**0.66−1.26**	**0.6**
**fixed effects model**	**421**	**211249**	**0.91**	**0.74−1.11**	**0.3**

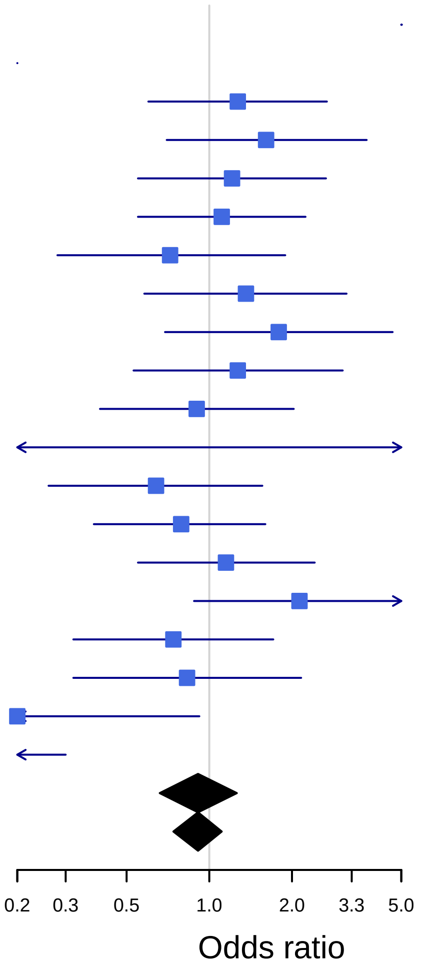

Propensity for Vbeta28

donor	V28	total TCRs	OR	CI	p value
1	26	13593	1	0.46−2.18	1
2	212	12567	0.93	0.71−1.22	0.6
3	18	14965	1.97	0.64−6.05	0.2
4	140	12387	1.17	0.83−1.63	0.4
5	96	8050	0.94	0.63−1.42	0.8
6	192	12302	0.75	0.56−1	0.05
7	98	9929	0.94	0.62−1.4	0.7
8	258	12477	1.14	0.89−1.46	0.3
9	20	13909	0.82	0.34−2	0.7
10	422	13074	1.24	1.01−1.51	0.04
11	242	12150	1.27	0.98−1.65	0.08
12	92	13082	1.27	0.83−1.95	0.3
13	132	14911	1.27	0.89−1.81	0.2
14	22	11142	5.29	1.55−18.02	0.008
15	16	10303	2.89	0.93−9.02	0.07
16	183	11252	0.78	0.58−1.05	0.1
17	135	8005	0.13	0.07−0.23	4e−12
18	45	7151	0.6	0.31−1.15	0.1
**random effects model**	**2349**	**211249**	**0.98**	**0.79−1.2**	**0.8**
**fixed effects model**	**2349**	**211249**	**1.02**	**0.93−1.11**	**0.7**

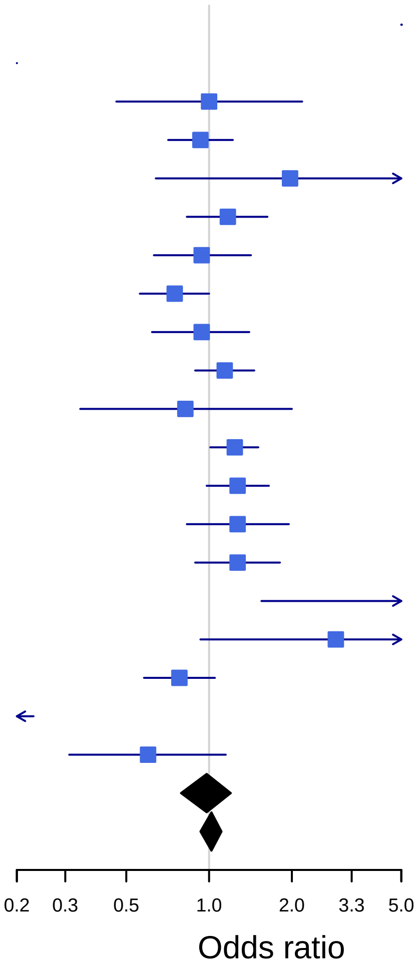

Propensity for Vbeta11.1

donor	V11.1	total TCRs	OR	CI	p value
1	155	13593	1	0.72−1.37	1
2	114	12567	0.71	0.49−1.04	0.08
3	114	14965	0.81	0.55−1.18	0.3
4	123	12387	1.58	1.1−2.28	0.01
5	75	8050	1.6	1−2.58	0.05
6	168	12302	0.72	0.53−0.98	0.04
7	104	9929	0.5	0.33−0.76	0.001
8	134	12477	1.11	0.79−1.58	0.5
9	149	13909	0.8	0.58−1.11	0.2
10	137	13074	1.56	1.09−2.21	0.01
11	82	12150	0.91	0.59−1.41	0.7
12	94	13082	1.33	0.87−2.03	0.2
13	204	14911	0.85	0.65−1.13	0.3
14	128	11142	0.84	0.59−1.2	0.3
15	102	10303	1.43	0.95−2.13	0.08
16	93	11252	0.97	0.64−1.46	0.9
17	49	8005	3.42	1.8−6.49	2e−04
18	62	7151	0.85	0.47−1.53	0.6
**random effects model**	**2087**	**211249**	**1.03**	**0.87−1.21**	**0.8**
**fixed effects model**	**2087**	**211249**	**0.99**	**0.91−1.08**	**0.8**

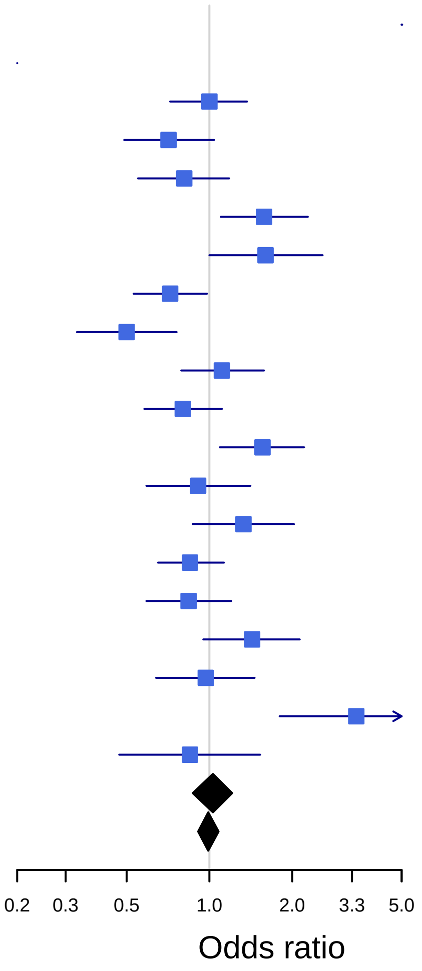

Propensity for Vbeta6.9

donor	V6.9	total TCRs	OR	CI	p value
1	44	13593	1.37	0.75−2.48	0.3
2	50	12567	1.3	0.74−2.3	0.4
3	81	14965	1.03	0.64−1.64	0.9
4	32	12387	0.7	0.34−1.41	0.3
5	39	8050	1.15	0.61−2.19	0.7
6	57	12302	1.24	0.73−2.11	0.4
7	50	9929	0.93	0.53−1.64	0.8
8	43	12477	0.73	0.39−1.34	0.3
9	48	13909	1.65	0.92−2.95	0.09
10	47	13074	0.72	0.4−1.29	0.3
11	16	12150	0.33	0.11−0.96	0.04
12	26	13082	1.42	0.61−3.29	0.4
13	70	14911	1.16	0.72−1.88	0.5
14	17	11142	4.09	1.16−14.35	0.03
15	22	10303	0.87	0.37−2.02	0.7
16	12	11252	1.78	0.53−5.98	0.4
17	3	8005	157668.69	0−6.46557105627354e+101	0.9
18	7	7151	0.57	0.11−2.98	0.5
**random effects model**	**664**	**211249**	**1.07**	**0.9−1.27**	**0.5**
**fixed effects model**	**664**	**211249**	**1.07**	**0.91−1.25**	**0.4**

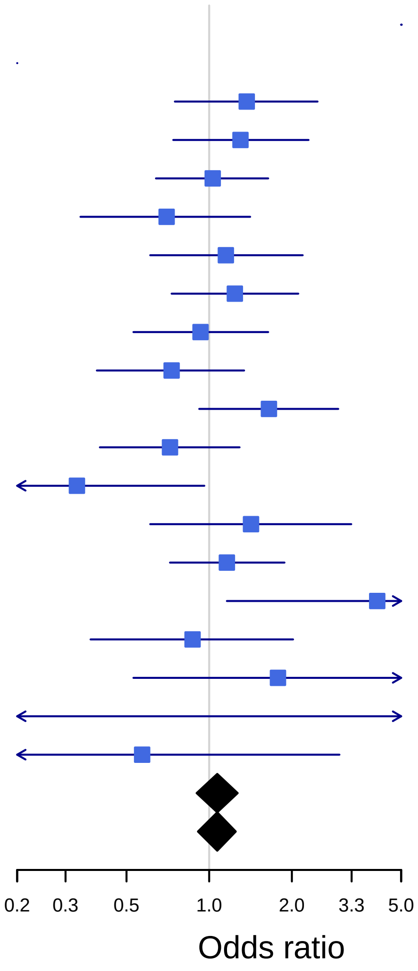

Propensity for Vbeta5.5

donor	V5.5	total TCRs	OR	CI	p value
1	618	13593	1.01	0.85−1.19	0.9
2	322	12567	1.17	0.94−1.46	0.2
3	382	14965	0.86	0.7−1.07	0.2
4	593	12387	0.97	0.82−1.15	0.7
5	301	8050	1.78	1.39−2.27	4e−06
6	449	12302	1.24	1.02−1.5	0.03
7	142	9929	1.03	0.74−1.45	0.8
8	290	12477	0.89	0.7−1.13	0.3
9	617	13909	1.47	1.25−1.73	4e−06
10	161	13074	1.12	0.82−1.54	0.5
11	94	12150	1.19	0.78−1.8	0.4
12	108	13082	1.75	1.15−2.66	0.009
13	180	14911	1.32	0.97−1.79	0.07
14	101	11142	1.73	1.13−2.65	0.01
15	123	10303	0.66	0.46−0.95	0.03
16	117	11252	1.63	1.11−2.39	0.01
17	94	8005	0.66	0.43−1.02	0.06
18	90	7151	0.96	0.57−1.6	0.9
**random effects model**	**4782**	**211249**	**1.14**	**1.01−1.29**	**0.04**
**fixed effects model**	**4782**	**211249**	**1.14**	**1.07−1.2**	**3e−05**

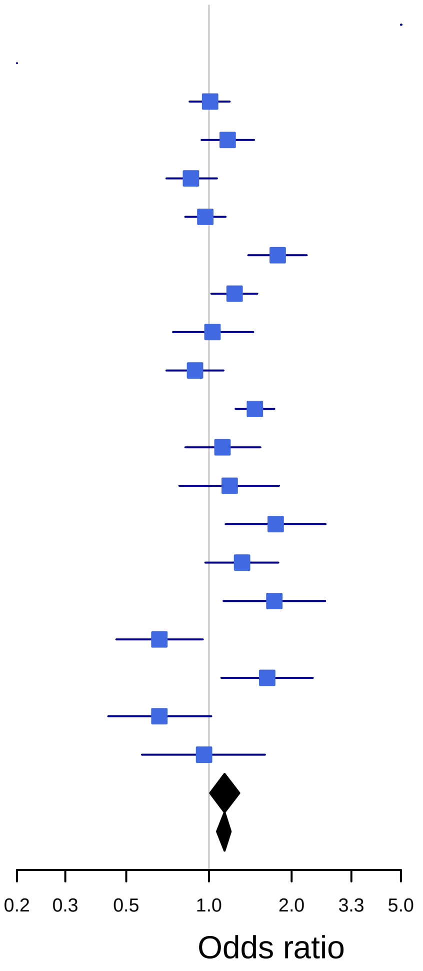

Propensity for Vbeta2

donor	V2	total TCRs	OR	CI	p value
1	309	13593	1.02	0.81−1.29	0.8
2	201	12567	1.34	1.01−1.78	0.05
3	202	14965	1.07	0.79−1.43	0.7
4	227	12387	1.39	1.06−1.82	0.02
5	150	8050	1.18	0.85−1.64	0.3
6	195	12302	0.83	0.62−1.1	0.2
7	136	9929	1.03	0.73−1.45	0.9
8	133	12477	0.94	0.66−1.32	0.7
9	189	13909	1.07	0.8−1.43	0.6
10	254	13074	1.6	1.23−2.08	5e−04
11	151	12150	0.95	0.69−1.32	0.8
12	123	13082	0.94	0.65−1.35	0.7
13	248	14911	1.14	0.88−1.48	0.3
14	145	11142	3.78	2.44−5.85	2e−09
15	230	10303	1.56	1.19−2.06	0.001
16	158	11252	2.11	1.49−2.99	2e−05
17	72	8005	0.1	0.04−0.24	1e−07
18	102	7151	0.54	0.35−0.83	0.004
**random effects model**	**3225**	**211249**	**1.12**	**0.92−1.35**	**0.3**
**fixed effects model**	**3225**	**211249**	**1.17**	**1.09−1.26**	**2e−05**

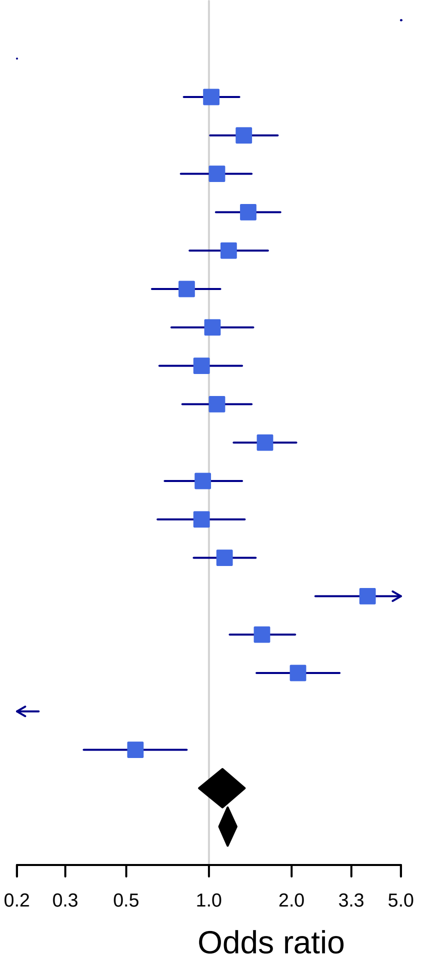

Propensity for Vbeta23.1

donor	V23.1	total TCRs	OR	CI	p value
1	20	13593	2.32	0.92−5.87	0.08
2	3	12567	1.81	0.16−20.63	0.6
3	7	14965	1.31	0.25−6.86	0.8
4	2	12387	0	0−4.15063041816719e+113	0.9
5	4	8050	0	0−4.79696330038694e+130	0.9
6	6	12302	3.88	0.45−33.82	0.2
7	10	9929	1.72	0.48−6.18	0.4
8	7	12477	0.4	0.08−2.09	0.3
9	10	13909	1.54	0.43−5.5	0.5
10	6	13074	0.36	0.06−2	0.2
11	9	12150	0.91	0.24−3.43	0.9
12	8	13082	2.49	0.5−12.44	0.3
13	5	14911	1.15	0.19−7.03	0.9
14	9	11142	4.89	0.61−39.41	0.1
15	7	10303	0.37	0.07−1.96	0.2
16	10	11252	3.47	0.73−16.56	0.1
17	4	8005	0.37	0.04−3.66	0.4
18	3	7151	0.07	0.01−0.81	0.03
**random effects model**	**130**	**211249**	**1.24**	**0.8−1.92**	**0.3**
**fixed effects model**	**130**	**211249**	**1.28**	**0.86−1.9**	**0.2**

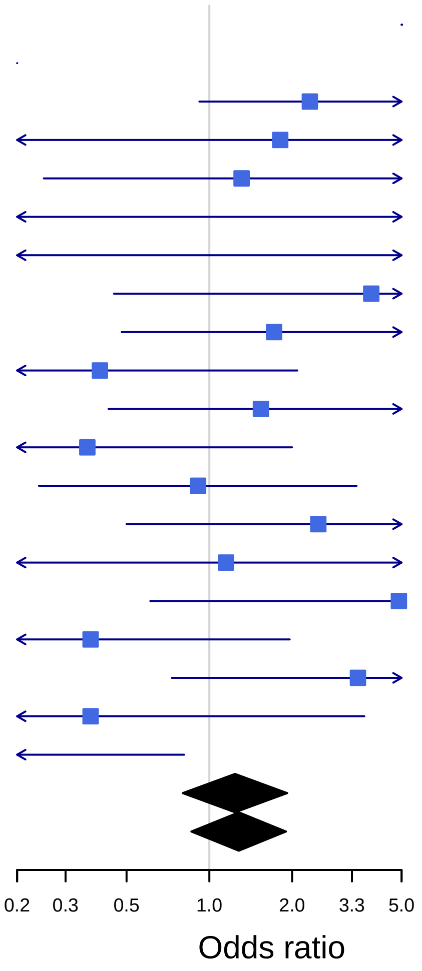

Propensity for Vbeta6.5

donor	V6.5	total TCRs	OR	CI	p value
1	944	13593	1.39	1.22−1.59	1e−06
2	1038	12567	1.21	1.06−1.37	0.004
3	1655	14965	0.96	0.86−1.07	0.4
4	822	12387	1.01	0.88−1.17	0.9
5	640	8050	1.05	0.89−1.23	0.6
6	1281	12302	1.13	1.01−1.28	0.03
7	946	9929	1.08	0.94−1.23	0.3
8	1072	12477	1.15	1.02−1.31	0.03
9	990	13909	1.61	1.41−1.83	2e−12
10	800	13074	1.76	1.51−2.05	5e−13
11	1092	12150	0.94	0.83−1.07	0.4
12	1141	13082	1.46	1.28−1.66	9e−09
13	1581	14911	1.22	1.1−1.36	3e−04
14	786	11142	1.79	1.53−2.1	4e−13
15	951	10303	1.45	1.26−1.67	1e−07
16	643	11252	0.82	0.7−0.96	0.02
17	372	8005	0.97	0.79−1.2	0.8
18	456	7151	1.89	1.41−2.54	2e−05
**random effects model**	**17210**	**211249**	**1.23**	**1.11−1.36**	**8e−05**
**fixed effects model**	**17210**	**211249**	**1.21**	**1.17−1.25**	**3e−30**

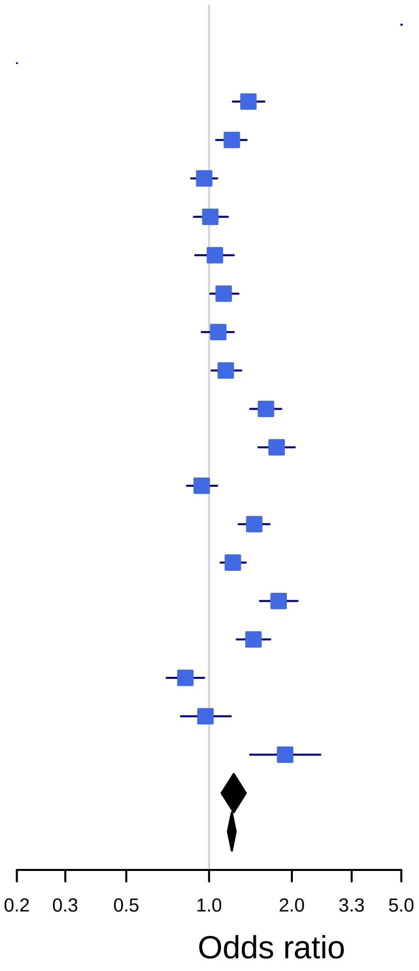

Propensity for Vbeta11.3

donor	V11.3	total TCRs	OR	CI	p value
1	20	13593	1.03	0.42−2.49	1
2	108	12567	1.79	1.2−2.66	0.004
3	167	14965	1.16	0.83−1.62	0.4
4	12	12387	1.95	0.58−6.59	0.3
5	8	8050	3.26	0.65−16.35	0.2
6	23	12302	0.56	0.24−1.3	0.2
7	17	9929	1.53	0.58−4.07	0.4
8	110	12477	1.45	0.99−2.14	0.06
9	17	13909	1.3	0.5−3.38	0.6
10	28	13074	1.43	0.67−3.07	0.4
11	10	12150	0.61	0.17−2.19	0.5
12	7	13082	4.72	0.56−39.63	0.2
13	16	14911	0.89	0.33−2.39	0.8
14	19	11142	1.89	0.71−5.02	0.2
15	14	10303	0.91	0.31−2.62	0.9
16	6	11252	235622.57	0−2.55225290376919e+116	0.9
17	6	8005	1.55	0.31−7.69	0.6
18	106	7151	0.68	0.44−1.04	0.08
**random effects model**	**694**	**211249**	**1.21**	**0.99−1.48**	**0.07**
**fixed effects model**	**694**	**211249**	**1.21**	**1.03−1.42**	**0.02**

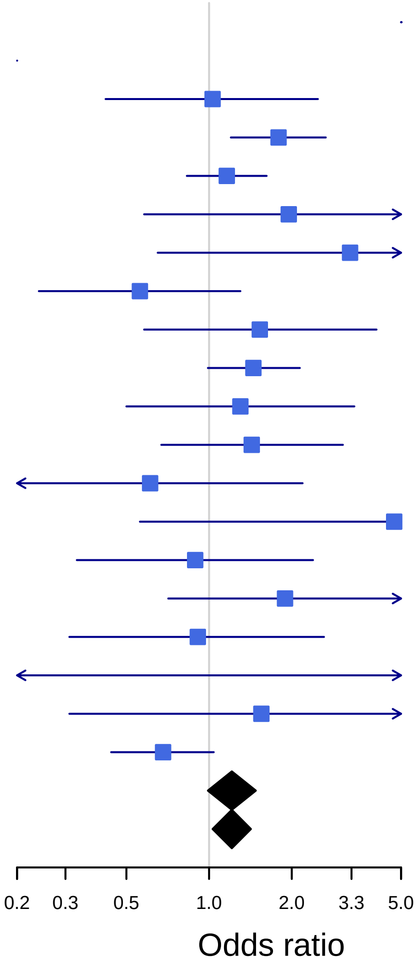

Propensity for Vbeta6.1

donor	V6.1	total TCRs	OR	CI	p value
1	340	13593	1.08	0.87−1.34	0.5
2	197	12567	1.86	1.38−2.51	5e−05
3	210	14965	1.03	0.77−1.37	0.9
4	364	12387	1.5	1.21−1.86	2e−04
5	189	8050	1.37	1.02−1.85	0.04
6	281	12302	1.75	1.36−2.25	1e−05
7	95	9929	1.07	0.71−1.61	0.8
8	163	12477	1.01	0.74−1.39	0.9
9	386	13909	1.4	1.14−1.72	0.001
10	156	13074	0.93	0.68−1.28	0.7
11	91	12150	1.22	0.79−1.87	0.4
12	115	13082	1.12	0.77−1.64	0.6
13	264	14911	1.05	0.82−1.35	0.7
14	101	11142	1.05	0.7−1.56	0.8
15	182	10303	1.52	1.12−2.06	0.008
16	109	11252	1.79	1.19−2.69	0.005
17	96	8005	1.38	0.92−2.08	0.1
18	74	7151	0.41	0.26−0.67	4e−04
**random effects model**	**3413**	**211249**	**1.22**	**1.07−1.39**	**0.003**
**fixed effects model**	**3413**	**211249**	**1.26**	**1.17−1.35**	**1e−10**

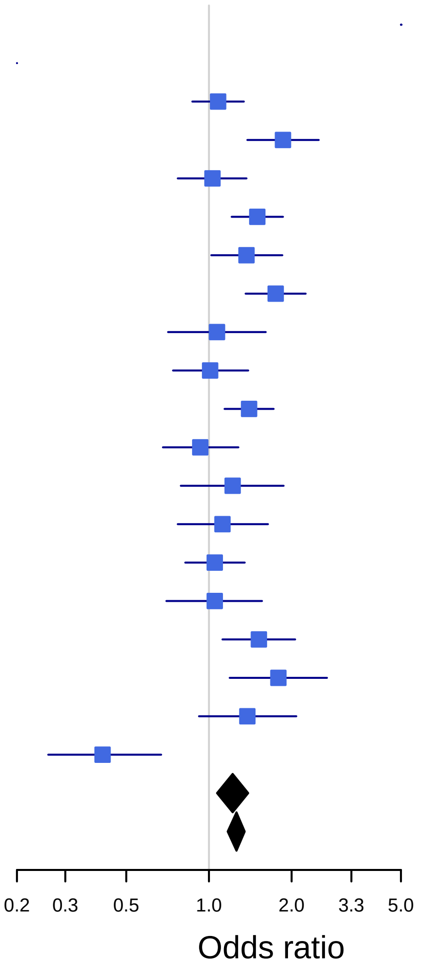

Propensity for Vbeta6.8

donor	V6.8	total TCRs	OR	CI	p value
1	2	13593	1.31	0.08−21.07	0.8
2	5	12567	0.72	0.12−4.35	0.7
3	6	14965	2.59	0.3−22.51	0.4
4	3	12387	0	0−1.91157127391665e+89	0.9
5	1	8050	26741.68	0−1.31962762223711e+106	0.9
6	4	12302	3.09	0.32−29.92	0.3
7	4	9929	2.98	0.3−29.14	0.3
8	4	12477	0.3	0.03−2.96	0.3
9	2	13909	1.05	0.06−16.89	1
10	8	13074	2.83	0.57−14.17	0.2
11	15	12150	1.11	0.39−3.17	0.8
12	5	13082	2.53	0.28−22.64	0.4
13	18	14911	2.06	0.73−5.82	0.2
14	16	11142	0.39	0.14−1.1	0.08
15	7	10303	6.33	0.76−52.82	0.09
16	7	11252	2.27	0.44−11.81	0.3
17	6	8005	1.17	0.23−5.93	0.8
18	5	7151	0.87	0.1−7.84	0.9
**random effects model**	**118**	**211249**	**1.3**	**0.86−1.97**	**0.2**
**fixed effects model**	**118**	**211249**	**1.3**	**0.86−1.97**	**0.2**

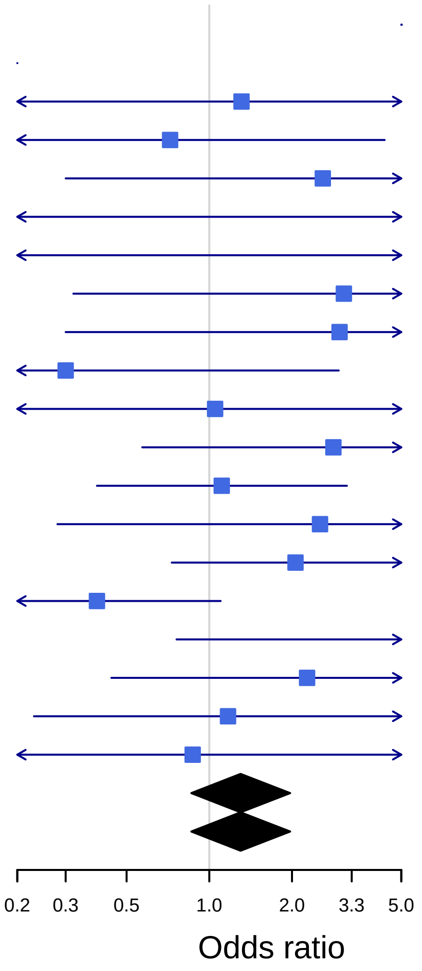

Propensity for Vbeta25.1

donor	V25.1	total TCRs	OR	CI	p value
1	46	13593	1.34	0.74−2.4	0.3
2	41	12567	0.58	0.31−1.09	0.09
3	37	14965	0.9	0.46−1.77	0.8
4	28	12387	1.51	0.71−3.21	0.3
5	17	8050	1.57	0.58−4.28	0.4
6	23	12302	0.62	0.27−1.43	0.3
7	35	9929	0.7	0.35−1.38	0.3
8	91	12477	2.37	1.49−3.78	3e−04
9	34	13909	1.78	0.89−3.56	0.1
10	33	13074	1.16	0.58−2.32	0.7
11	26	12150	1.5	0.66−3.37	0.3
12	25	13082	0.9	0.41−2.01	0.8
13	53	14911	1.81	1.01−3.23	0.05
14	29	11142	4.04	1.53−10.68	0.005
15	23	10303	2.16	0.88−5.29	0.09
16	23	11252	3.34	1.23−9.05	0.02
17	20	8005	0.23	0.07−0.78	0.02
18	20	7151	0.73	0.26−2.03	0.5
**random effects model**	**604**	**211249**	**1.26**	**0.95−1.67**	**0.1**
**fixed effects model**	**604**	**211249**	**1.31**	**1.1−1.55**	**0.002**

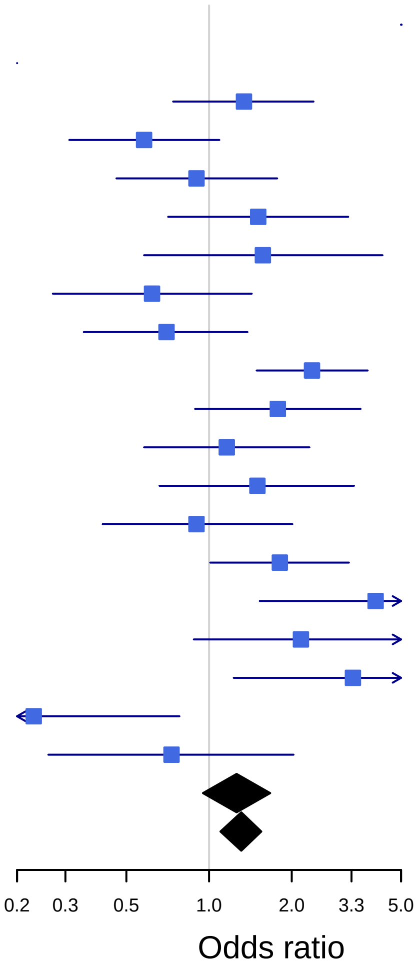

Propensity for Vbeta6.2

donor	V6.2	total TCRs	OR	CI	p value
1	574	13593	1.19	1−1.4	0.05
2	365	12567	1.98	1.58−2.47	2e−09
3	1018	14965	1.35	1.17−1.55	4e−05
4	290	12387	0.92	0.72−1.16	0.5
5	297	8050	1.5	1.18−1.91	9e−04
6	476	12302	1.59	1.32−1.93	2e−06
7	332	9929	1.68	1.34−2.11	7e−06
8	671	12477	1.3	1.11−1.53	0.001
9	325	13909	1.32	1.06−1.65	0.01
10	564	13074	1.39	1.16−1.65	2e−04
11	384	12150	1.37	1.1−1.69	0.004
12	506	13082	0.91	0.76−1.09	0.3
13	305	14911	1.55	1.22−1.97	3e−04
14	492	11142	0.79	0.65−0.94	0.01
15	262	10303	1.06	0.83−1.36	0.6
16	414	11252	1.67	1.35−2.05	1e−06
17	456	8005	2.88	2.35−3.53	2e−24
18	337	7151	1.35	1−1.82	0.05
**random effects model**	**8068**	**211249**	**1.37**	**1.19−1.57**	**9e−06**
**fixed effects model**	**8068**	**211249**	**1.35**	**1.29−1.41**	**5e−36**

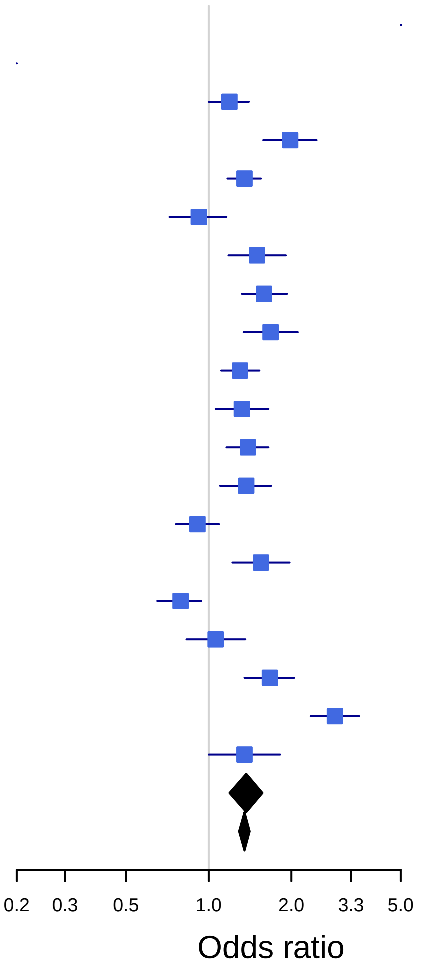

Propensity for Vbeta7.3

donor	V7.3	total TCRs	OR	CI	p value
1	4	13593	0.45	0.05−4.32	0.5
2	7	12567	1.44	0.32−6.47	0.6
3	3	14965	1.22	0.11−13.52	0.9
4	6	12387	2.12	0.38−11.79	0.4
5	4	8050	2.88	0.29−28.24	0.4
6	3	12302	0	0−2.94466733227061e+91	0.9
7	3	9929	1.89	0.17−20.86	0.6
8	27	12477	3.08	1.29−7.36	0.01
9	5	13909	0.76	0.13−4.58	0.8
10	4	13074	2.27	0.23−22.17	0.5
11	3	12150	0.37	0.03−4.09	0.4
12	4	13082	1.72	0.17−17.12	0.6
13	9	14911	1.04	0.27−3.98	1
14	2	11142	83136.62	0−2.34500592470351e+123	0.9
15	5	10303	0.6	0.1−3.65	0.6
16	6	11252	1.58	0.29−8.78	0.6
17	2	8005	0	0−5.35622561014301e+112	0.9
18	5	7151	0.85	0.09−7.71	0.9
**random effects model**	**102**	**211249**	**1.5**	**0.97−2.34**	**0.07**
**fixed effects model**	**102**	**211249**	**1.5**	**0.97−2.34**	**0.07**

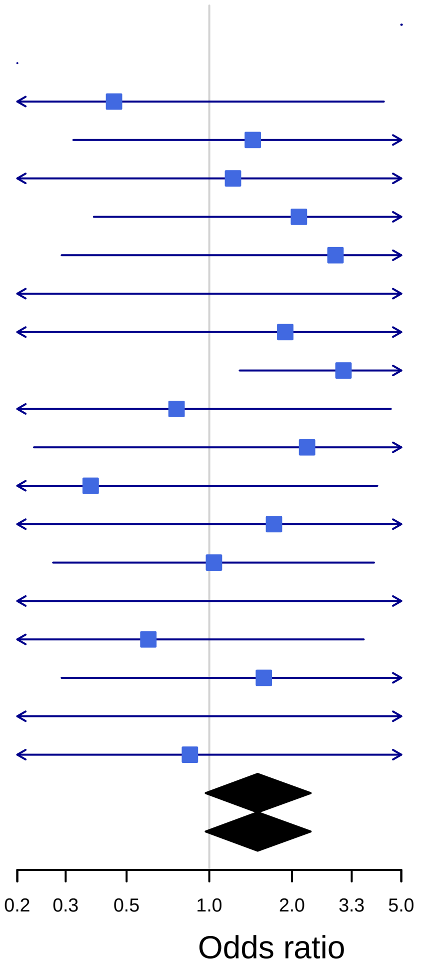

Propensity for Vbeta6.6

donor	V6.6	total TCRs	OR	CI	p value
1	639	13593	1.37	1.17−1.61	1e−04
2	555	12567	1.36	1.14−1.61	6e−04
3	735	14965	1.17	1−1.38	0.06
4	528	12387	1.55	1.29−1.85	2e−06
5	447	8050	1.34	1.1−1.63	0.004
6	646	12302	1.47	1.25−1.74	4e−06
7	484	9929	1.13	0.94−1.35	0.2
8	557	12477	1.19	1−1.42	0.05
9	630	13909	1.49	1.26−1.75	2e−06
10	558	13074	1.82	1.51−2.18	1e−10
11	464	12150	1.17	0.97−1.42	0.1
12	554	13082	1.37	1.14−1.64	7e−04
13	927	14911	1.28	1.11−1.46	5e−04
14	406	11142	1.66	1.34−2.05	3e−06
15	520	10303	1.58	1.31−1.9	1e−06
16	436	11252	1.87	1.52−2.3	2e−09
17	253	8005	1.58	1.23−2.04	4e−04
18	295	7151	1.06	0.79−1.43	0.7
**random effects model**	**9634**	**211249**	**1.4**	**1.3−1.5**	**1e−20**
**fixed effects model**	**9634**	**211249**	**1.39**	**1.33−1.45**	**2e−51**

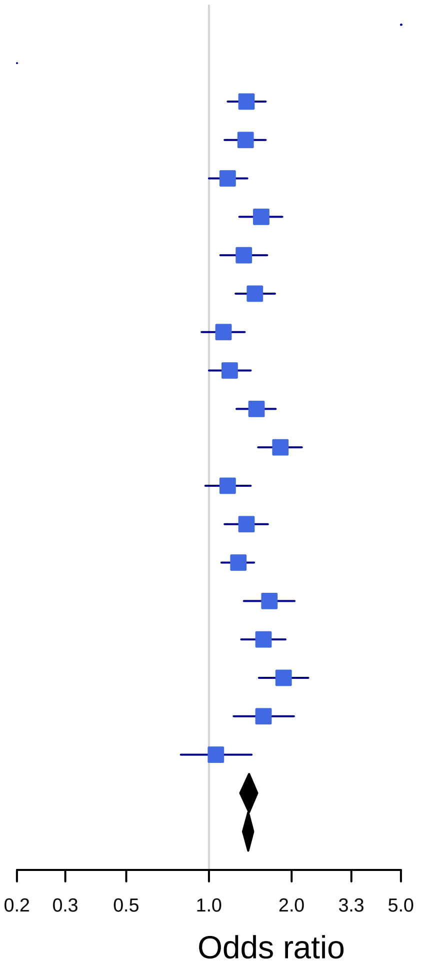

Propensity for Vbeta5.4

donor	V5.4	total TCRs	OR	CI	p value
1	35	13593	1.16	0.59−2.26	0.7
2	27	12567	0.86	0.4−1.84	0.7
3	70	14965	3.6	1.78−7.27	4e−04
4	47	12387	1.1	0.62−1.98	0.7
5	25	8050	2.78	1.1−7	0.03
6	47	12302	0.73	0.41−1.3	0.3
7	144	9929	0.93	0.67−1.3	0.7
8	43	12477	1.24	0.68−2.28	0.5
9	58	13909	1.02	0.61−1.72	0.9
10	159	13074	1.38	0.99−1.91	0.05
11	80	12150	1.54	0.96−2.45	0.07
12	103	13082	3.01	1.85−4.88	8e−06
13	232	14911	1.56	1.19−2.06	0.001
14	66	11142	1.9	1.12−3.23	0.02
15	82	10303	1.5	0.95−2.35	0.08
16	125	11252	2.36	1.58−3.53	3e−05
17	74	8005	0.46	0.28−0.77	0.003
18	80	7151	1.12	0.64−1.98	0.7
**random effects model**	**1497**	**211249**	**1.37**	**1.1−1.7**	**0.005**
**fixed effects model**	**1497**	**211249**	**1.37**	**1.23−1.53**	**2e−08**

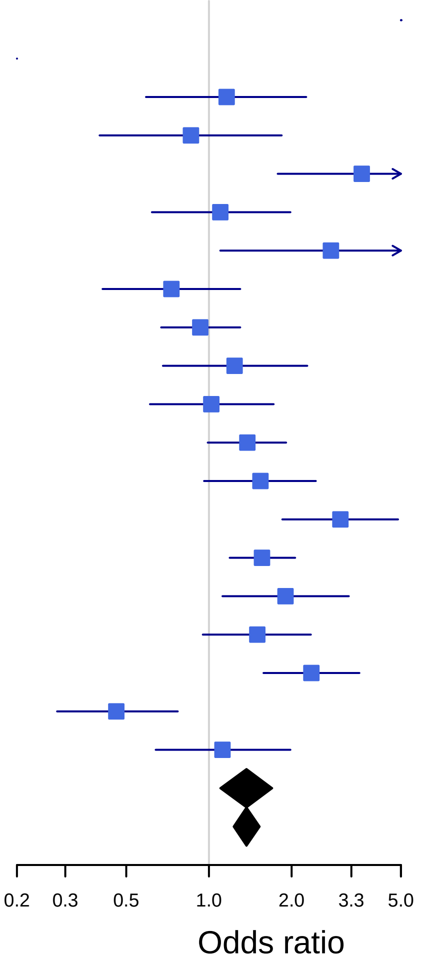

Propensity for Vbeta10.3

donor	V10.3	total TCRs	OR	CI	p value
1	215	13593	0.98	0.74−1.28	0.9
2	236	12567	1.67	1.27−2.18	2e−04
3	315	14965	1.35	1.05−1.73	0.02
4	206	12387	1.86	1.39−2.48	3e−05
5	126	8050	0.95	0.67−1.36	0.8
6	307	12302	1.25	1−1.58	0.06
7	216	9929	1.07	0.82−1.41	0.6
8	395	12477	2.01	1.62−2.49	2e−10
9	351	13909	1.1	0.89−1.36	0.4
10	256	13074	2.21	1.68−2.91	2e−08
11	288	12150	1.13	0.89−1.43	0.3
12	313	13082	2.41	1.85−3.15	7e−11
13	391	14911	1.7	1.37−2.11	1e−06
14	293	11142	2.56	1.95−3.35	1e−11
15	208	10303	1.63	1.22−2.18	9e−04
16	324	11252	2.13	1.67−2.72	1e−09
17	113	8005	0.51	0.34−0.77	0.001
18	141	7151	1.1	0.71−1.69	0.7
**random effects model**	**4694**	**211249**	**1.45**	**1.22−1.71**	**2e−05**
**fixed effects model**	**4694**	**211249**	**1.5**	**1.41−1.6**	**1e−37**

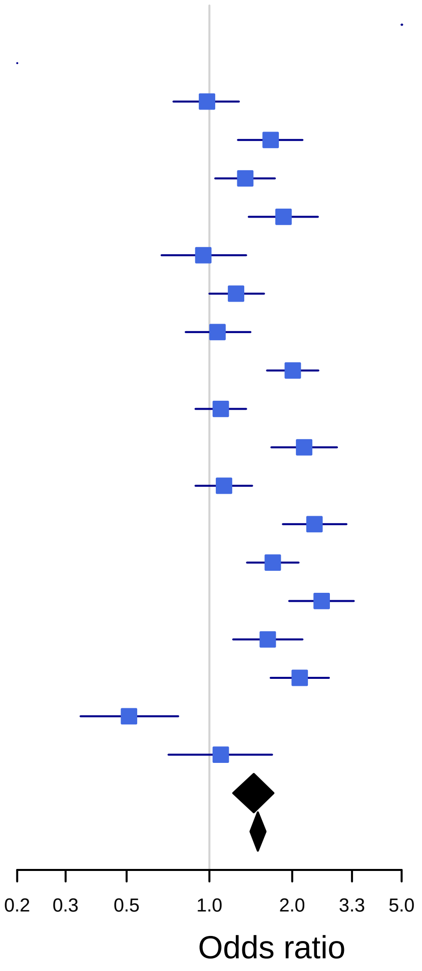

Propensity for Vbeta12.3

donor	V12.3	total TCRs	OR	CI	p value
1	486	13593	1.87	1.55−2.25	5e−11
2	451	12567	1.43	1.18−1.73	3e−04
3	907	14965	1.82	1.55−2.13	3e−13
4	566	12387	2.36	1.96−2.84	6e−20
5	411	8050	1.54	1.25−1.9	4e−05
6	496	12302	2.31	1.89−2.82	2e−16
7	468	9929	0.87	0.72−1.05	0.2
8	565	12477	1.85	1.55−2.22	2e−11
9	598	13909	1.76	1.48−2.08	6e−11
10	429	13074	2.19	1.76−2.72	1e−12
11	212	12150	1.47	1.1−1.97	0.008
12	280	13082	1.51	1.17−1.96	0.002
13	672	14911	1.49	1.27−1.75	2e−06
14	232	11142	7.61	4.93−11.75	5e−20
15	456	10303	1.8	1.47−2.2	1e−08
16	494	11252	0.55	0.46−0.66	3e−10
17	140	8005	0.51	0.35−0.73	3e−04
18	248	7151	0.68	0.51−0.92	0.01
**random effects model**	**8111**	**211249**	**1.53**	**1.23−1.89**	**1e−04**
**fixed effects model**	**8111**	**211249**	**1.53**	**1.46−1.61**	**3e−67**

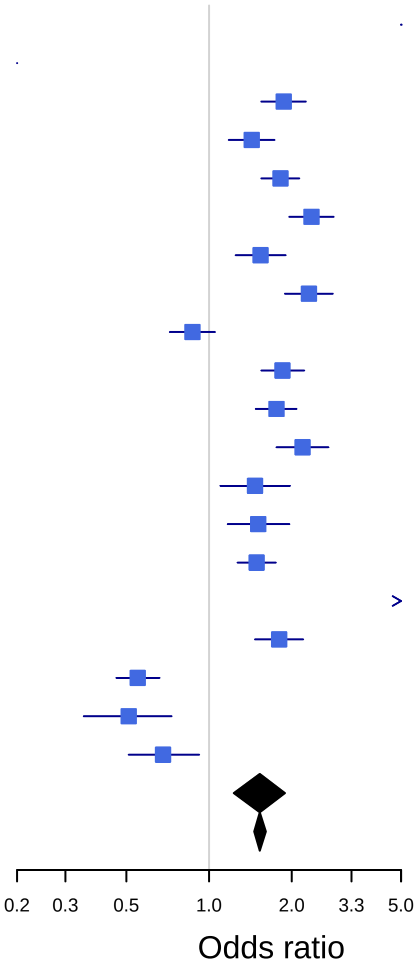

Propensity for Vbeta20.1

donor	V20.1	total TCRs	OR	CI	p value
1	509	13593	1.4	1.17−1.67	2e−04
2	686	12567	2.47	2.08−2.93	2e−25
3	750	14965	2.15	1.79−2.59	3e−16
4	687	12387	3.01	2.53−3.59	2e−35
5	366	8050	3.18	2.48−4.08	7e−20
6	411	12302	3.79	2.96−4.85	4e−26
7	489	9929	2.37	1.95−2.89	8e−18
8	578	12477	1.98	1.66−2.37	5e−14
9	611	13909	2.61	2.19−3.12	3e−26
10	630	13074	2.67	2.22−3.22	2e−25
11	390	12150	2.31	1.83−2.91	2e−12
12	397	13082	2.67	2.09−3.4	2e−15
13	573	14911	2.26	1.87−2.72	3e−17
14	246	11142	12.78	7.56−21.61	2e−21
15	332	10303	1.95	1.54−2.47	3e−08
16	604	11252	4.36	3.52−5.4	3e−41
17	162	8005	0.07	0.03−0.13	9e−14
18	215	7151	0.54	0.4−0.73	4e−05
**random effects model**	**8636**	**211249**	**2.17**	**1.73−2.72**	**2e−11**
**fixed effects model**	**8636**	**211249**	**2.34**	**2.23−2.46**	**<1e−100**

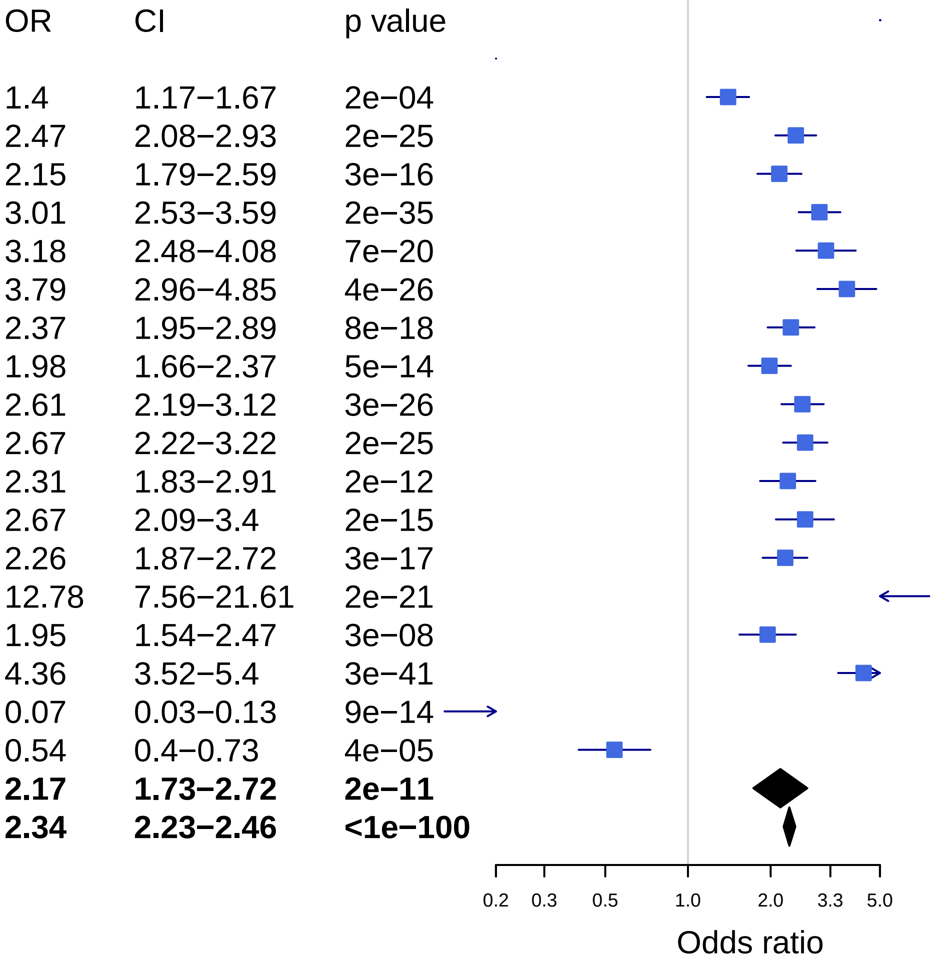

Propensity for Vbeta7.2

donor	V7.2	total TCRs	OR	CI	p value
1	118	13593	1.9	1.31−2.76	7e−04
2	83	12567	2.11	1.32−3.39	0.002
3	267	14965	2.79	2.01−3.89	1e−09
4	83	12387	2.51	1.55−4.05	2e−04
5	127	8050	4.62	2.9−7.36	1e−10
6	126	12302	1.99	1.36−2.91	4e−04
7	155	9929	2.89	2.02−4.14	7e−09
8	166	12477	4.85	3.22−7.32	5e−14
9	91	13909	1.76	1.15−2.69	0.009
10	143	13074	4.34	2.79−6.76	8e−11
11	73	12150	3.7	2.02−6.76	2e−05
12	41	13082	1.92	0.95−3.85	0.07
13	66	14911	1.8	1.07−3.04	0.03
14	81	11142	21.5	6.77−68.29	2e−07
15	40	10303	1.91	0.96−3.78	0.06
16	92	11252	3.46	2.08−5.75	2e−06
17	70	8005	0.13	0.06−0.28	3e−07
18	70	7151	1.14	0.61−2.13	0.7
**random effects model**	**1892**	**211249**	**2.38**	**1.78−3.19**	**7e−09**
**fixed effects model**	**1892**	**211249**	**2.51**	**2.24−2.81**	**1e−58**

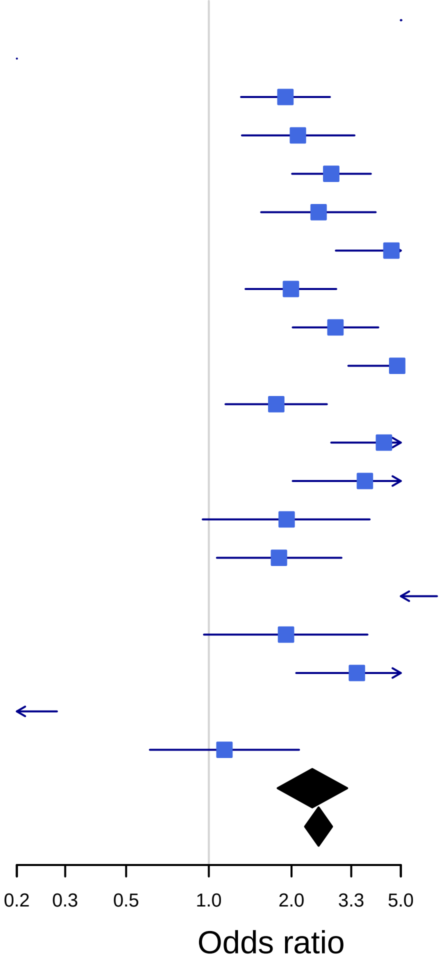

Propensity for Vbeta5.1

donor	V5.1	total TCRs	OR	CI	p value
1	636	13593	1.97	1.67−2.32	6e−16
2	499	12567	2.44	2−2.97	1e−18
3	607	14965	2.75	2.22−3.42	3e−20
4	538	12387	1.5	1.25−1.79	8e−06
5	439	8050	4.03	3.16−5.13	2e−29
6	688	12302	5.41	4.38−6.69	5e−55
7	527	9929	2.57	2.12−3.11	9e−22
8	295	12477	2.61	2.01−3.38	4e−13
9	552	13909	2.14	1.79−2.56	1e−16
10	450	13074	2.14	1.74−2.63	7e−13
11	494	12150	2.08	1.7−2.54	1e−12
12	300	13082	4.09	2.99−5.61	2e−18
13	671	14911	4.3	3.5−5.28	4e−44
14	448	11142	13.29	9.04−19.54	2e−39
15	546	10303	2.32	1.92−2.81	4e−18
16	602	11252	3.9	3.17−4.79	8e−38
17	224	8005	0.1	0.06−0.17	4e−20
18	352	7151	1.68	1.23−2.3	0.001
**random effects model**	**8868**	**211249**	**2.49**	**1.93−3.21**	**3e−12**
**fixed effects model**	**8868**	**211249**	**2.56**	**2.44−2.7**	**<1e−100**

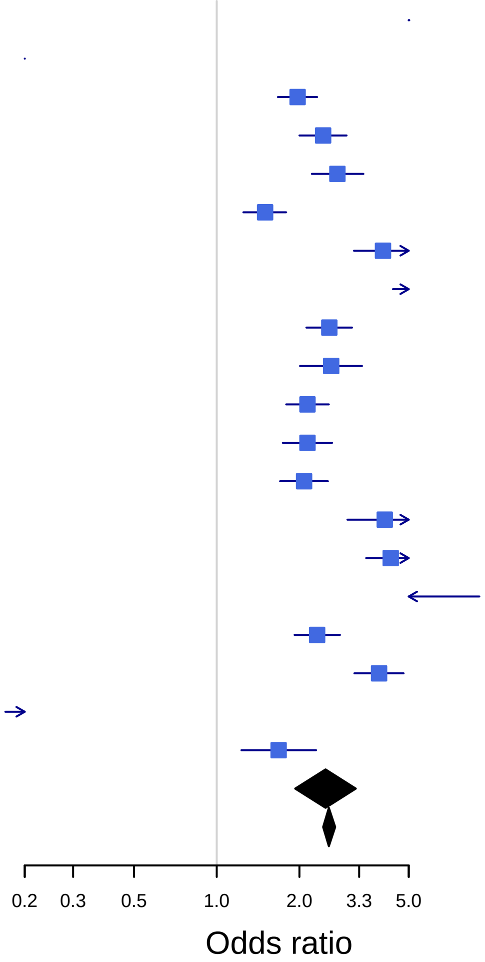

Propensity for Vbeta18

donor	V18	total TCRs	OR	CI	p value
1	239	13593	6.14	4.35−8.69	9e−25
2	237	12567	5.61	3.91−8.06	9e−21
3	380	14965	5.35	3.77−7.6	7e−21
4	214	12387	3.79	2.73−5.28	3e−15
5	165	8050	5.49	3.55−8.51	2e−14
6	432	12302	6.2	4.68−8.22	7e−37
7	213	9929	4.58	3.23−6.5	1e−17
8	208	12477	6.25	4.21−9.3	1e−19
9	276	13909	5.55	4.03−7.64	7e−26
10	110	13074	1.82	1.21−2.74	0.004
11	140	12150	2.06	1.41−3.01	2e−04
12	256	13082	3.41	2.45−4.73	3e−13
13	366	14911	3.37	2.6−4.35	2e−20
14	226	11142	3.58	2.55−5.03	2e−13
15	273	10303	6.96	4.79−10.1	2e−24
16	254	11252	6.48	4.44−9.47	4e−22
17	489	8005	18.31	12.87−26.04	9e−59
18	115	7151	2.31	1.24−4.33	0.009
**random effects model**	**4593**	**211249**	**4.71**	**3.73−5.95**	**1e−38**
**fixed effects model**	**4593**	**211249**	**4.78**	**4.4−5.2**	**<1e−100**

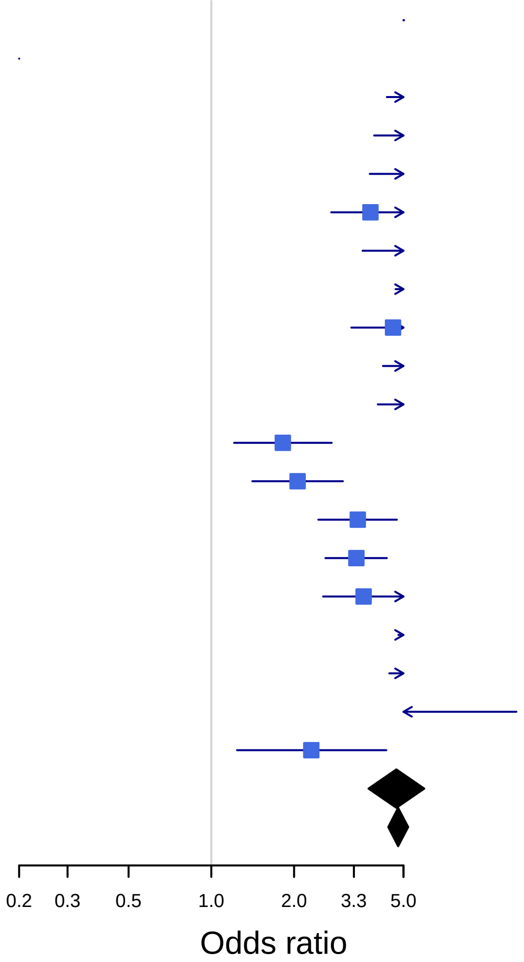

Supplement: S2 File — (PDF) [file pone.0140815.s002.pdf]
