# Supplementary material for: Somatic Variation of T-Cell Receptor Genes Strongly Associate with HLA Class Restriction
Source: PLoS One. 2015 Oct 30;10(10):e0140815. doi: 10.1371/journal.pone.0140815 (PMC4627806; doi:10.1371/journal.pone.0140815)

Propensity for Valpha1.2

donor	V1.2	total TCRs	OR	CI	p value
1	189	8250	0.27	0.11−0.66	0.004
2	137	7609	0.09	0.03−0.29	4e−05
3	87	10674	0.15	0.08−0.28	1e−09
4	123	10324	0.35	0.23−0.55	3e−06
5	152	11216	0.17	0.11−0.26	1e−17
**random effects model**	**688**	**48073**	**0.2**	**0.13−0.31**	**4e−13**
**fixed effects model**	**688**	**48073**	**0.21**	**0.17−0.27**	**9e−34**

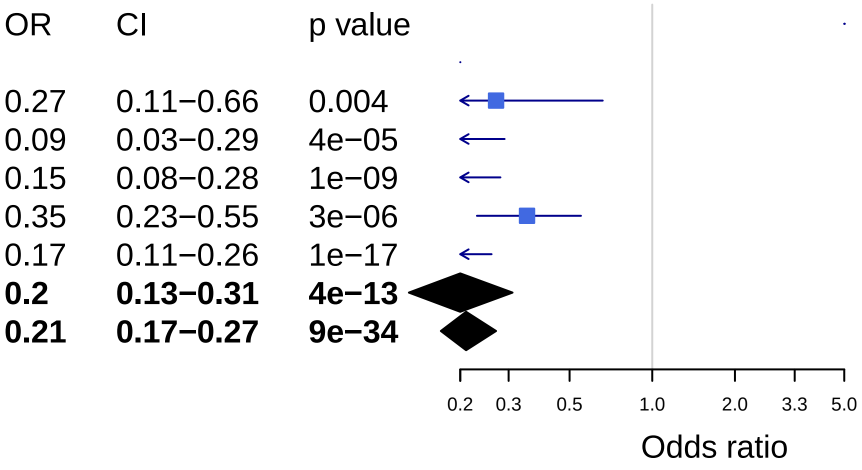

Propensity for Valpha4

donor	V4	total TCRs	OR	CI	p value
1	612	8250	0.42	0.28−0.62	1e−05
2	606	7609	0.39	0.3−0.52	5e−11
3	704	10674	0.34	0.29−0.4	2e−36
4	739	10324	0.2	0.16−0.25	1e−52
5	627	11216	0.25	0.21−0.3	7e−51
**random effects model**	**3288**	**48073**	**0.3**	**0.23−0.39**	**5e−20**
**fixed effects model**	**3288**	**48073**	**0.29**	**0.26−0.32**	**<1e−100**

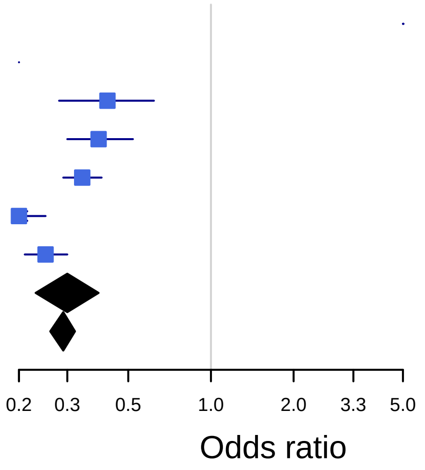

Propensity for Valpha24

donor	V24	total TCRs	OR	CI	p value
1	67	8250	1.11	0.5−2.47	0.8
2	62	7609	0.89	0.46−1.72	0.7
3	182	10674	0.43	0.32−0.59	1e−07
4	64	10324	0.35	0.19−0.63	4e−04
5	135	11216	0.28	0.19−0.41	4e−11
**random effects model**	**510**	**48073**	**0.49**	**0.31−0.76**	**0.001**
**fixed effects model**	**510**	**48073**	**0.42**	**0.34−0.52**	**9e−17**

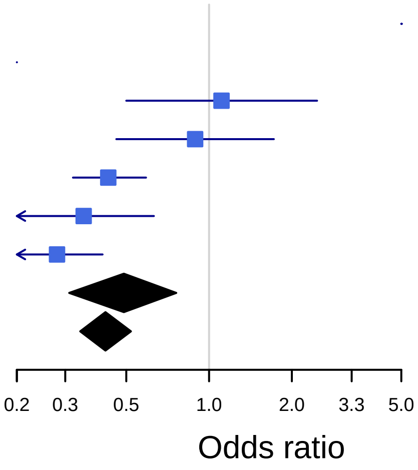

Propensity for Valpha3

donor	V3	total TCRs	OR	CI	p value
1	73	8250	0.58	0.21−1.6	0.3
2	40	7609	0.72	0.3−1.74	0.5
3	57	10674	1.01	0.59−1.72	1
4	85	10324	0.28	0.16−0.49	8e−06
5	87	11216	0.33	0.21−0.52	2e−06
**random effects model**	**342**	**48073**	**0.5**	**0.29−0.88**	**0.02**
**fixed effects model**	**342**	**48073**	**0.47**	**0.36−0.62**	**5e−08**

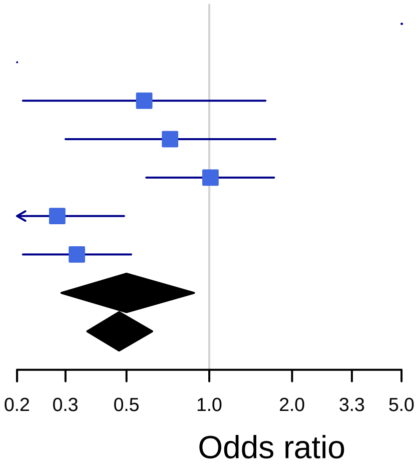

Propensity for Valpha21

donor	V21	total TCRs	OR	CI	p value
1	292	8250	1.04	0.7−1.56	0.8
2	282	7609	0.41	0.27−0.61	1e−05
3	467	10674	0.55	0.45−0.66	7e−10
4	292	10324	0.9	0.71−1.14	0.4
5	411	11216	0.22	0.18−0.28	4e−38
**random effects model**	**1744**	**48073**	**0.54**	**0.31−0.94**	**0.03**
**fixed effects model**	**1744**	**48073**	**0.5**	**0.45−0.56**	**4e−32**

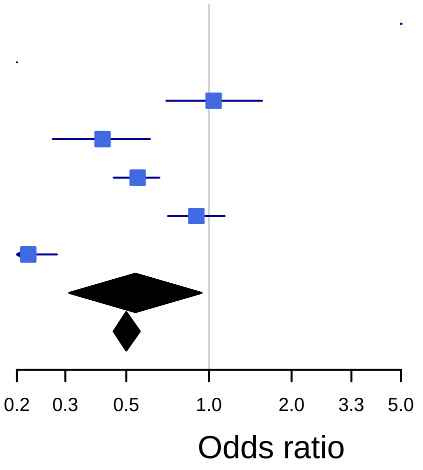

Propensity for Valpha12.2

donor	V12.2	total TCRs	OR	CI	p value
1	208	8250	0.24	0.1−0.58	0.002
2	204	7609	0.43	0.26−0.69	5e−04
3	235	10674	0.56	0.43−0.74	3e−05
4	370	10324	0.52	0.41−0.65	3e−08
5	287	11216	0.46	0.36−0.58	3e−10
**random effects model**	**1304**	**48073**	**0.49**	**0.42−0.57**	**2e−21**
**fixed effects model**	**1304**	**48073**	**0.49**	**0.43−0.56**	**8e−25**

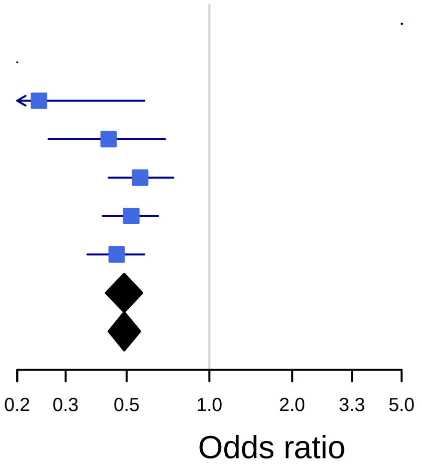

Propensity for Valpha38.1

donor	V38.1	total TCRs	OR	CI	p value
1	266	8250	0.73	0.45−1.18	0.2
2	115	7609	0.76	0.46−1.25	0.3
3	381	10674	0.66	0.54−0.82	1e−04
4	600	10324	0.51	0.43−0.61	9e−13
5	394	11216	0.38	0.31−0.47	6e−19
**random effects model**	**1756**	**48073**	**0.56**	**0.44−0.72**	**8e−06**
**fixed effects model**	**1756**	**48073**	**0.53**	**0.47−0.59**	**3e−30**

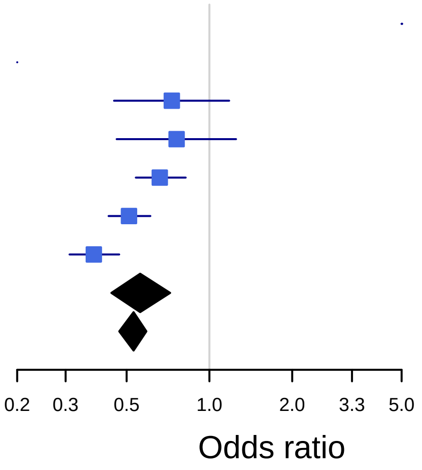

Propensity for Valpha30

donor	V30	total TCRs	OR	CI	p value
1	179	8250	0.99	0.59−1.67	1
2	227	7609	0.58	0.39−0.85	0.005
3	286	10674	0.45	0.35−0.57	2e−10
4	192	10324	0.59	0.43−0.8	8e−04
5	173	11216	0.6	0.44−0.82	0.001
**random effects model**	**1057**	**48073**	**0.58**	**0.47−0.72**	**9e−07**
**fixed effects model**	**1057**	**48073**	**0.56**	**0.48−0.65**	**4e−15**

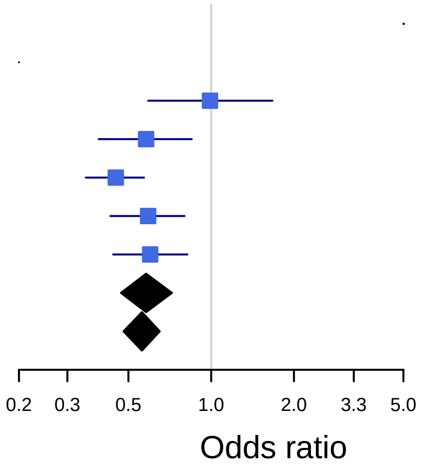

Propensity for Valpha8.1

donor	V8.1	total TCRs	OR	CI	p value
1	92	8250	0.67	0.29−1.54	0.3
2	84	7609	0.89	0.51−1.55	0.7
3	108	10674	1.03	0.7−1.51	0.9
4	202	10324	1.02	0.77−1.36	0.9
5	325	11216	0.35	0.27−0.44	2e−18
**random effects model**	**811**	**48073**	**0.73**	**0.42−1.28**	**0.3**
**fixed effects model**	**811**	**48073**	**0.63**	**0.54−0.73**	**3e−09**

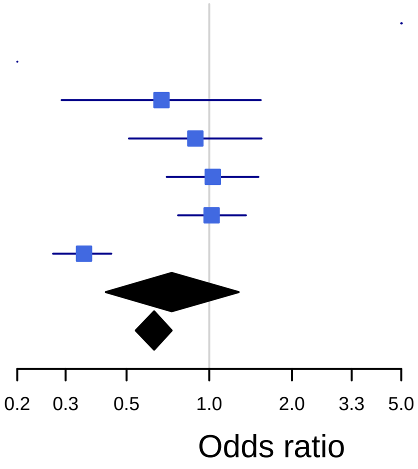

Propensity for Valpha8.2

donor	V8.2	total TCRs	OR	CI	p value
1	59	8250	1.93	0.97−3.87	0.06
2	77	7609	0.88	0.49−1.6	0.7
3	83	10674	0.55	0.35−0.86	0.009
4	95	10324	0.78	0.51−1.19	0.3
5	169	11216	0.48	0.35−0.65	4e−06
**random effects model**	**483**	**48073**	**0.76**	**0.5−1.15**	**0.2**
**fixed effects model**	**483**	**48073**	**0.66**	**0.54−0.8**	**3e−05**

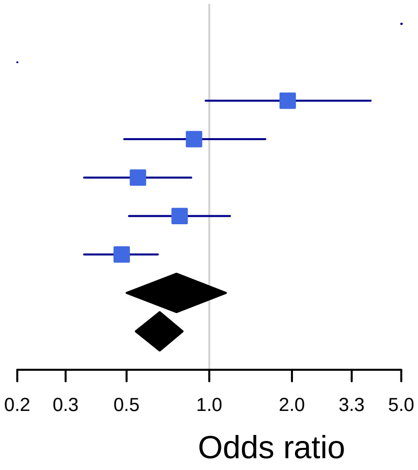

Propensity for Valpha12.1

donor	V12.1	total TCRs	OR	CI	p value
1	364	8250	0.47	0.29−0.77	0.002
2	450	7609	0.4	0.29−0.55	4e−08
3	366	10674	0.64	0.52−0.79	4e−05
4	361	10324	0.83	0.66−1.03	0.09
5	808	11216	0.72	0.62−0.83	1e−05
**random effects model**	**2349**	**48073**	**0.62**	**0.5−0.77**	**2e−05**
**fixed effects model**	**2349**	**48073**	**0.67**	**0.61−0.74**	**3e−15**

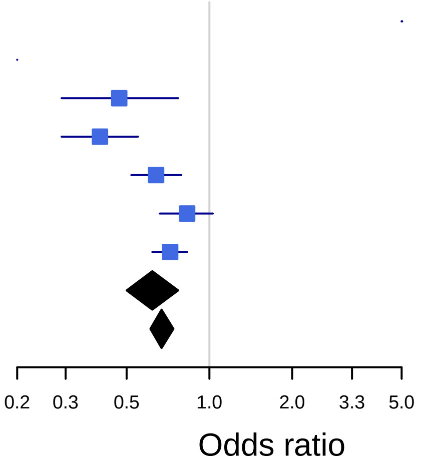

Propensity for Valpha8

donor	V8	total TCRs	OR	CI	p value
1	315	8250	0.87	0.57−1.34	0.5
2	191	7609	1.23	0.86−1.74	0.3
3	562	10674	0.89	0.74−1.06	0.2
4	700	10324	0.56	0.47−0.66	2e−11
5	526	11216	0.61	0.51−0.73	1e−07
**random effects model**	**2294**	**48073**	**0.78**	**0.6−1.01**	**0.06**
**fixed effects model**	**2294**	**48073**	**0.71**	**0.65−0.78**	**2e−12**

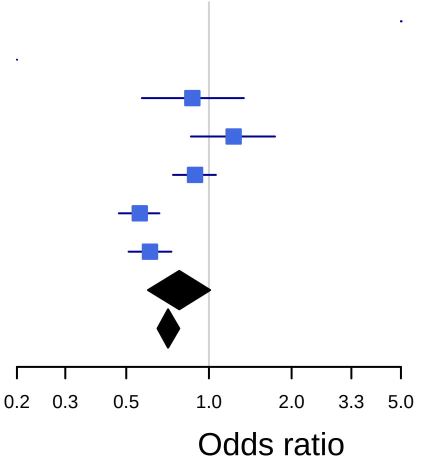

Propensity for Valpha17

donor	V17	total TCRs	OR	CI	p value
1	522	8250	0.45	0.29−0.68	2e−04
2	534	7609	0.91	0.73−1.15	0.4
3	574	10674	0.76	0.64−0.91	0.002
4	554	10324	0.75	0.63−0.9	0.002
5	764	11216	0.63	0.54−0.74	3e−09
**random effects model**	**2948**	**48073**	**0.71**	**0.61−0.84**	**4e−05**
**fixed effects model**	**2948**	**48073**	**0.72**	**0.66−0.78**	**5e−14**

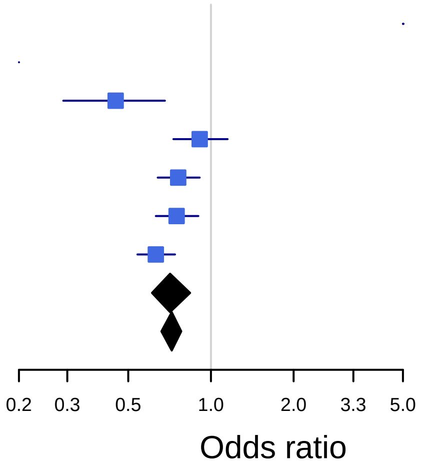

Propensity for Valpha27

donor	V27	total TCRs	OR	CI	p value
1	256	8250	0.85	0.54−1.34	0.5
2	210	7609	0.82	0.57−1.18	0.3
3	416	10674	0.92	0.75−1.12	0.4
4	396	10324	0.99	0.8−1.21	0.9
5	460	11216	0.59	0.49−0.71	6e−08
**random effects model**	**1738**	**48073**	**0.82**	**0.65−1.02**	**0.08**
**fixed effects model**	**1738**	**48073**	**0.8**	**0.72−0.89**	**6e−05**

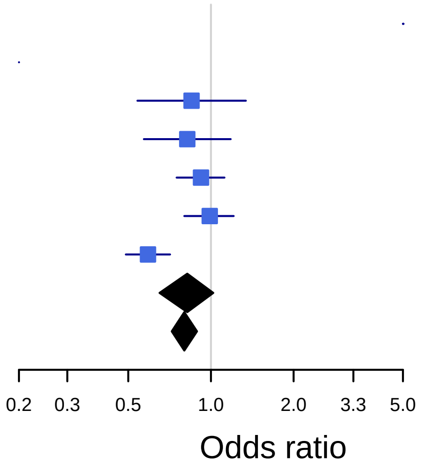

Propensity for Valpha12.3

donor	V12.3	total TCRs	OR	CI	p value
1	336	8250	0.64	0.41−0.99	0.05
2	266	7609	0.87	0.63−1.19	0.4
3	519	10674	0.97	0.81−1.16	0.7
4	467	10324	0.95	0.78−1.15	0.6
5	443	11216	1.1	0.9−1.35	0.3
**random effects model**	**2031**	**48073**	**0.95**	**0.84−1.08**	**0.4**
**fixed effects model**	**2031**	**48073**	**0.96**	**0.87−1.07**	**0.5**

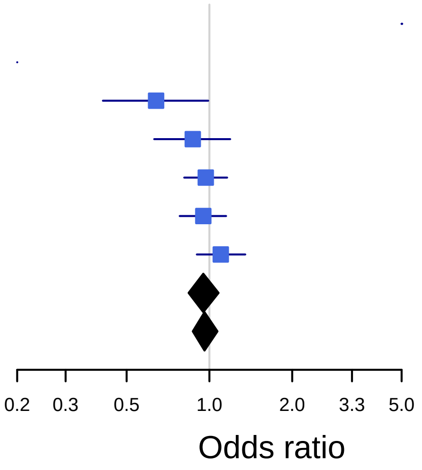

Propensity for Valpha8.6

donor	V8.6	total TCRs	OR	CI	p value
1	110	8250	1.63	0.94−2.8	0.08
2	96	7609	1.37	0.86−2.19	0.2
3	174	10674	1.36	0.99−1.86	0.06
4	179	10324	1.08	0.8−1.46	0.6
5	298	11216	0.82	0.65−1.04	0.1
**random effects model**	**857**	**48073**	**1.16**	**0.9−1.49**	**0.3**
**fixed effects model**	**857**	**48073**	**1.08**	**0.93−1.25**	**0.3**

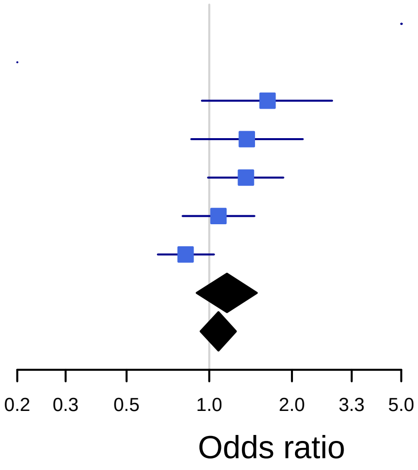

Propensity for Valpha13.2

donor	V13.2	total TCRs	OR	CI	p value
1	963	8250	0.52	0.39−0.69	1e−05
2	679	7609	1.36	1.13−1.64	0.001
3	875	10674	0.87	0.75−1	0.05
4	713	10324	0.85	0.72−0.99	0.04
5	591	11216	3.19	2.58−3.95	2e−26
**random effects model**	**3821**	**48073**	**1.11**	**0.67−1.84**	**0.7**
**fixed effects model**	**3821**	**48073**	**1.09**	**1−1.18**	**0.04**

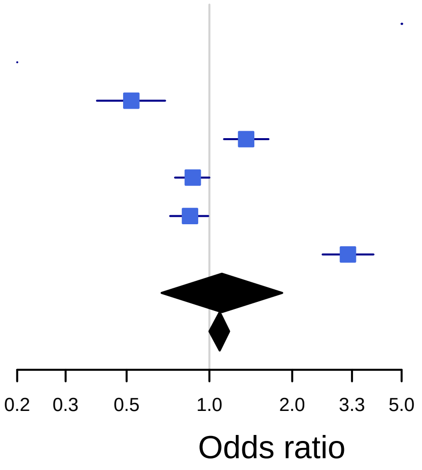

Propensity for Valpha5

donor	V5	total TCRs	OR	CI	p value
1	291	8250	1.45	1.01−2.08	0.05
2	629	7609	0.62	0.49−0.78	5e−05
3	647	10674	0.84	0.71−0.99	0.03
4	591	10324	1.02	0.86−1.2	0.9
5	398	11216	5.03	3.67−6.89	1e−23
**random effects model**	**2556**	**48073**	**1.3**	**0.74−2.27**	**0.4**
**fixed effects model**	**2556**	**48073**	**1.04**	**0.94−1.14**	**0.5**

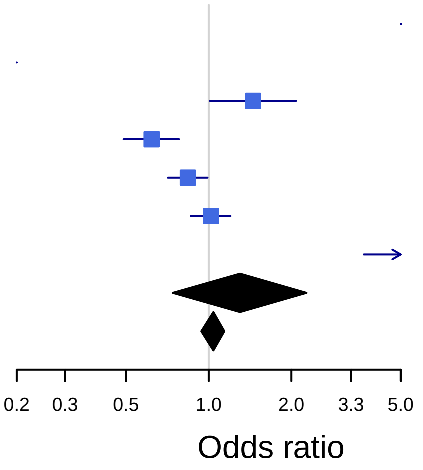

Propensity for Valpha25

donor	V25	total TCRs	OR	CI	p value
1	127	8250	1.36	0.79−2.32	0.3
2	128	7609	1.19	0.78−1.8	0.4
3	235	10674	1.13	0.86−1.47	0.4
4	151	10324	1.48	1.07−2.05	0.02
5	175	11216	0.79	0.58−1.07	0.1
**random effects model**	**816**	**48073**	**1.14**	**0.9−1.43**	**0.3**
**fixed effects model**	**816**	**48073**	**1.12**	**0.96−1.3**	**0.1**

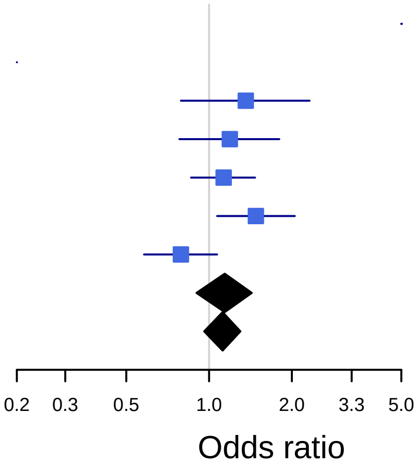

Propensity for Valpha8.4

donor	V8.4	total TCRs	OR	CI	p value
1	87	8250	1.87	1.02−3.42	0.04
2	129	7609	1.44	0.96−2.16	0.08
3	198	10674	1.35	1.01−1.81	0.05
4	170	10324	1.17	0.86−1.59	0.3
5	262	11216	0.89	0.69−1.14	0.4
**random effects model**	**846**	**48073**	**1.22**	**0.97−1.54**	**0.08**
**fixed effects model**	**846**	**48073**	**1.17**	**1.01−1.35**	**0.04**

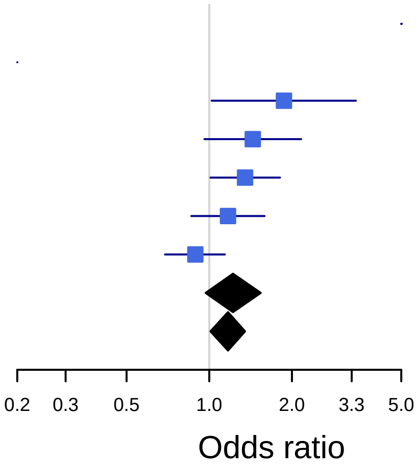

Propensity for Valpha26.2

donor	V26.2	total TCRs	OR	CI	p value
1	410	8250	0.74	0.5−1.09	0.1
2	387	7609	1.45	1.15−1.84	0.002
3	281	10674	1.03	0.81−1.32	0.8
4	311	10324	1.44	1.15−1.81	0.002
5	127	11216	1.28	0.87−1.87	0.2
**random effects model**	**1516**	**48073**	**1.18**	**0.95−1.48**	**0.1**
**fixed effects model**	**1516**	**48073**	**1.23**	**1.09−1.39**	**0.001**

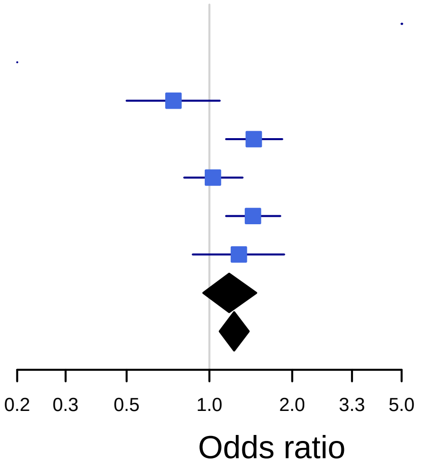

Propensity for Valpha35

donor	V35	total TCRs	OR	CI	p value
1	378	8250	0.63	0.41−0.97	0.03
2	258	7609	1	0.74−1.37	1
3	407	10674	1.19	0.96−1.46	0.1
4	367	10324	1.6	1.29−1.98	2e−05
5	181	11216	4.08	2.66−6.26	1e−10
**random effects model**	**1591**	**48073**	**1.37**	**0.88−2.11**	**0.2**
**fixed effects model**	**1591**	**48073**	**1.34**	**1.18−1.51**	**3e−06**

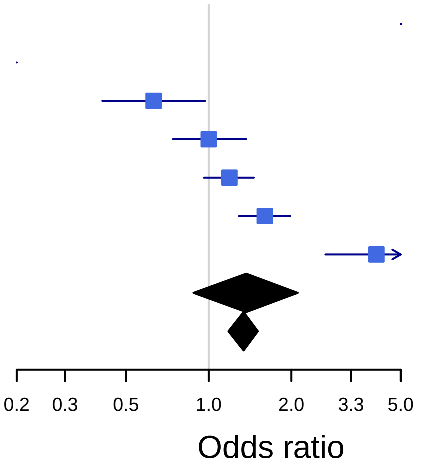

Propensity for Valpha8.3

donor	V8.3	total TCRs	OR	CI	p value
1	224	8250	1.52	1.02−2.26	0.04
2	214	7609	1.14	0.81−1.59	0.5
3	301	10674	1.69	1.32−2.16	3e−05
4	293	10324	1.51	1.2−1.92	6e−04
5	347	11216	1.78	1.4−2.27	3e−06
**random effects model**	**1379**	**48073**	**1.55**	**1.35−1.79**	**5e−10**
**fixed effects model**	**1379**	**48073**	**1.56**	**1.38−1.77**	**8e−13**

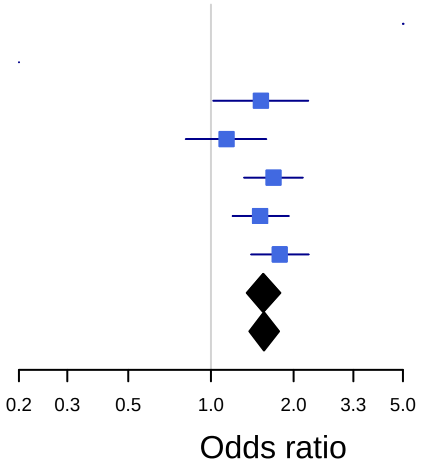

Propensity for Valpha6

donor	V6	total TCRs	OR	CI	p value
1	124	8250	1.37	0.8−2.34	0.3
2	203	7609	1.47	1.07−2.03	0.02
3	294	10674	2.55	1.95−3.34	1e−11
4	261	10324	1.69	1.32−2.18	4e−05
5	269	11216	1.74	1.33−2.29	6e−05
**random effects model**	**1151**	**48073**	**1.78**	**1.44−2.19**	**6e−08**
**fixed effects model**	**1151**	**48073**	**1.82**	**1.59−2.08**	**2e−18**

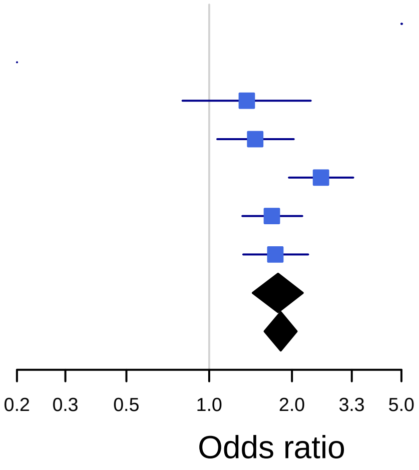

Propensity for Valpha7

donor	V7	total TCRs	OR	CI	p value
1	34	8250	2.43	1.04−5.69	0.04
2	39	7609	1.6	0.79−3.22	0.2
3	64	10674	1.56	0.92−2.64	0.1
4	89	10324	2.25	1.45−3.49	3e−04
5	84	11216	3.23	1.81−5.78	8e−05
**random effects model**	**310**	**48073**	**2.13**	**1.64−2.76**	**1e−08**
**fixed effects model**	**310**	**48073**	**2.13**	**1.65−2.75**	**8e−09**

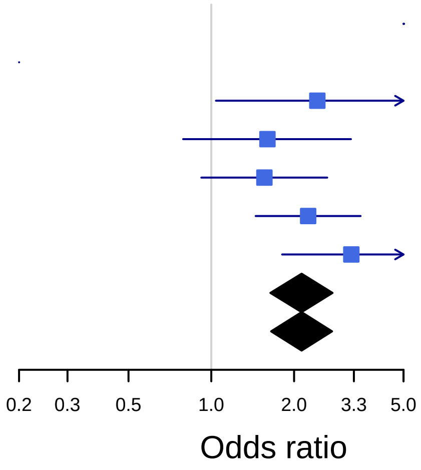

Propensity for Valpha40

donor	V40	total TCRs	OR	CI	p value
1	11	8250	0.99	0.12−7.89	1
2	10	7609	0.94	0.19−4.63	0.9
3	31	10674	3.88	1.48−10.21	0.006
4	21	10324	1.95	0.8−4.8	0.1
5	21	11216	2.06	0.74−5.71	0.2
**random effects model**	**94**	**48073**	**2.13**	**1.28−3.54**	**0.003**
**fixed effects model**	**94**	**48073**	**2.13**	**1.28−3.54**	**0.003**

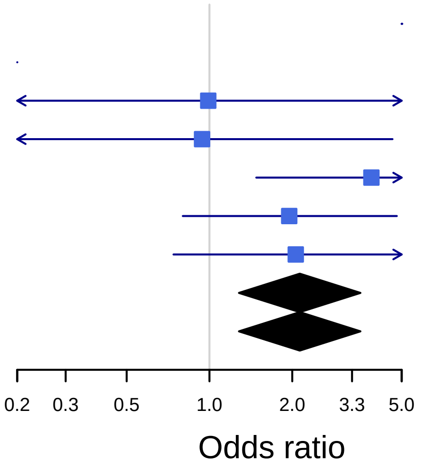

Propensity for Valpha2

donor	V2	total TCRs	OR	CI	p value
1	651	8250	3.9	3.17−4.8	3e−38
2	597	7609	1.99	1.64−2.41	2e−12
3	664	10674	2.15	1.8−2.56	8e−18
4	729	10324	2.58	2.19−3.03	2e−30
5	915	11216	4.32	3.59−5.19	6e−55
**random effects model**	**3556**	**48073**	**2.84**	**2.11−3.83**	**7e−12**
**fixed effects model**	**3556**	**48073**	**2.79**	**2.57−3.03**	**<1e−100**

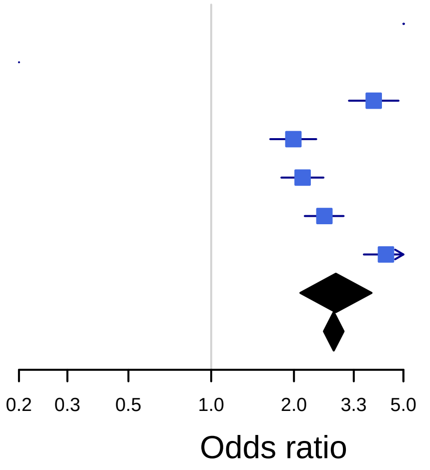

Propensity for Valpha9.2

donor	V9.2	total TCRs	OR	CI	p value
1	11	8250	3.17	0.82−12.29	0.09
2	34	7609	3.24	1.61−6.5	0.001
3	27	10674	2.53	1.06−6.05	0.04
4	42	10324	6.12	2.7−13.9	1e−05
5	79	11216	1.96	1.17−3.28	0.01
**random effects model**	**193**	**48073**	**2.95**	**1.96−4.44**	**2e−07**
**fixed effects model**	**193**	**48073**	**2.81**	**2.02−3.92**	**8e−10**

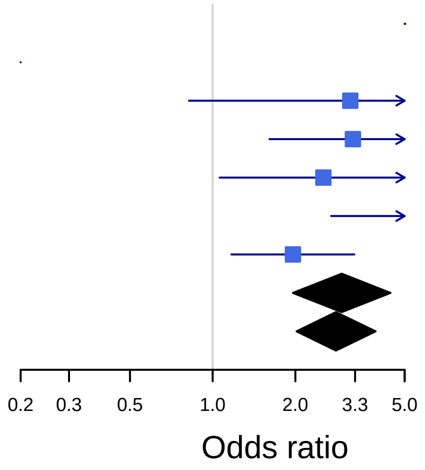

Propensity for Valpha26.1

donor	V26.1	total TCRs	OR	CI	p value
1	787	8250	1.88	1.52−2.34	9e−09
2	512	7609	2.93	2.43−3.55	2e−28
3	1359	10674	4.01	3.47−4.62	7e−81
4	1026	10324	4.02	3.47−4.66	4e−77
5	1538	11216	4.86	4.17−5.66	2e−91
**random effects model**	**5222**	**48073**	**3.39**	**2.56−4.5**	**2e−17**
**fixed effects model**	**5222**	**48073**	**3.67**	**3.41−3.95**	**<1e−100**

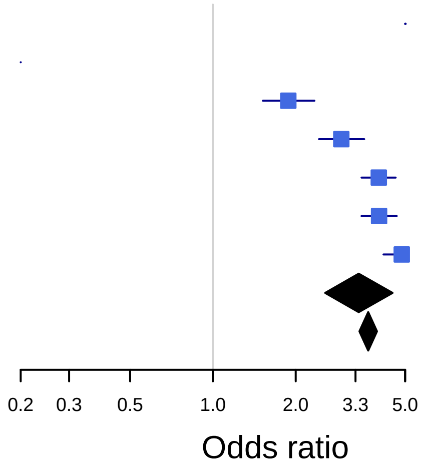

Supplement: S3 File — (PDF) [file pone.0140815.s003.pdf]
